# Cellular mechanisms for cargo delivery and polarity maintenance at different polar domains in plant cells

**DOI:** 10.1038/celldisc.2016.18

**Published:** 2016-07-19

**Authors:** Łukasz Łangowski, Krzysztof Wabnik, Hongjiang Li, Steffen Vanneste, Satoshi Naramoto, Hirokazu Tanaka, Jiří Friml

**Affiliations:** 1 Department of Plant Systems Biology, VIB, Ghent, Belgium; 2 Department of Plant Biotechnology and Bioinformatics, Ghent University, Ghent, Belgium; 3 Institute of Science and Technology Austria (IST Austria), Klosterneuburg, Austria; 4 Department of Biological Sciences, Graduate School of Science, University of Tokyo, Tokyo, Japan; 5 Department of Life Sciences, International Christian University, Tokyo, Japan; 6 Department of Biological Science, Graduate School of Science, University of Osaka, Osaka, Japan; 7 Mendel Centre for Plant Genomics and Proteomics, Central European Institute of Technology (CEITEC), Masaryk University, Brno, Czech Republic

**Keywords:** protein trafficking, polar recycling, polar secretion, lateral diffusion, protein clustering, protein dynamics modeling

## Abstract

The asymmetric localization of proteins in the plasma membrane domains of eukaryotic cells is a fundamental manifestation of cell polarity that is central to multicellular organization and developmental patterning. In plants, the mechanisms underlying the polar localization of cargo proteins are still largely unknown and appear to be fundamentally distinct from those operating in mammals. Here, we present a systematic, quantitative comparative analysis of the polar delivery and subcellular localization of proteins that characterize distinct polar plasma membrane domains in plant cells. The combination of microscopic analyses and computational modeling revealed a mechanistic framework common to diverse polar cargos and underlying the establishment and maintenance of apical, basal, and lateral polar domains in plant cells. This mechanism depends on the polar secretion, constitutive endocytic recycling, and restricted lateral diffusion of cargos within the plasma membrane. Moreover, our observations suggest that polar cargo distribution involves the individual protein potential to form clusters within the plasma membrane and interact with the extracellular matrix. Our observations provide insights into the shared cellular mechanisms of polar cargo delivery and polarity maintenance in plant cells.

## Introduction

The asymmetric distribution of proteins is a prerequisite of many cellular processes such as cell division, intracellular communication, nutrient transport, tissue morphogenesis, and cell differentiation [1–3]. In plants, the polar localization of proteins delineates up to four distinct polar plasma membrane (PM) domains that are designated as apical (shootward), basal (rootward), outer (peripheral), and inner (central) [1]. However, the mechanisms underlying the delivery of membrane cargos to these different domains and how polarity is maintained remain poorly understood, in part due to the lack of obvious homologs of the mammalian polarity regulators [4, 5]. For example, proteinaceous structures such as tight junctions that physically separate the apical from the baso-lateral PM domains in mammalian epithelial cells cannot be detected in most plant cell types. Only endodermis cells can become encircled by lignin bands [6] called Casparian strips, which act as physical barriers on the radial and transverse walls to restrict the exchange of water and nutrients [7]. Although this polar band can separate outer and inner polar domains in endodermal cells, it does not interfere with the apical and basal polarization [8]. Together, these findings suggest that plants have acquired a unique strategy to generate and maintain the subcellular polar distribution of proteins in the PM [9–11].

Well-characterized polarly localized proteins in plants are the PM-localized PIN-FORMED (PIN) auxin efflux carriers [12] that mainly mark the apical and basal polar domains [13–15]. PIN proteins constitutively (re)cycle between PM and endosomal compartments, while maintaining a seemingly static polar localization at the PM [16, 17]. Therefore, rigorous control mechanisms based on constrained lateral diffusion, super-polar exocytosis, and local endocytosis have been postulated to contribute to the maintenance of the PIN polarity [18]. Consistently with this model, perturbations in PIN trafficking at the endocytosis level have been correlated with strong defects in the PIN polarization [16, 19, 20]. In addition, distinct PIN secretion/recycling pathways have been identified that require the activity of various ADP-ribosylation factor guanine-nucleotide exchange factors such as GNOM [21, 22] and others [23–28]. Finally, the plant extracellular matrix, the cell wall seems to participate in the maintenance of the PIN protein polarization [29], presumably by constraining lateral diffusion in the PM [30] or by a still unknown mechanism [31, 32]. Besides these cellular polarity determinants, PIN proteins possess protein-intrinsic signals, such as sequence-specific factors [33]; some of them related to the PIN phosphorylation status [34–38].

Compared with the apical and basal PIN polarization mechanisms, very little is known about the processes underlying protein deposition to the outer and inner polar domains. Yet, it is becoming increasingly clear that these lateral polar domains are crucial for multiple aspects of the plant's life, as indicated by the localization to these lateral polar domains of multiple nutrient transporters, pathogen-related and other crucial proteins such as nodulation26-like intrinsic protein 5;1 (NIP5;1), boron 4 (BOR4) and BOR1 transporters, ECERIFERUM 5/ATP-binding cassette G12) (CER5/ABCG12), desperato (DSO/ABCG11), polar auxin transport inhibitor-sensitive 1/pleiotropic drug resistance 9 (PIS1/PDR9/ABCG37), and penetration 3/PDR8/ABCG36 (PEN3/PDR8/ABCG36) [4]. Not many genetic or pharmacological manipulations that affect the PIN polarization also impair these protein polarities [8, 39–41], suggesting a distinct underlying mechanism for polar targeting to and polarity maintenance at these polar domains.

Here, we compared systematically the mechanisms that support polar delivery and polarity maintenance of cargos targeted to apical, basal, outer, and inner domains. By combining quantitative microscopy and model simulations, we dissected the role of secretion, lateral diffusion, and endocytic recycling processes in the positioning of PM proteins at different polar domains of plant cells.

## Results

### Evaluation of cargo polarity at the apical, basal, outer, and inner polar domains

**Figure 1:**
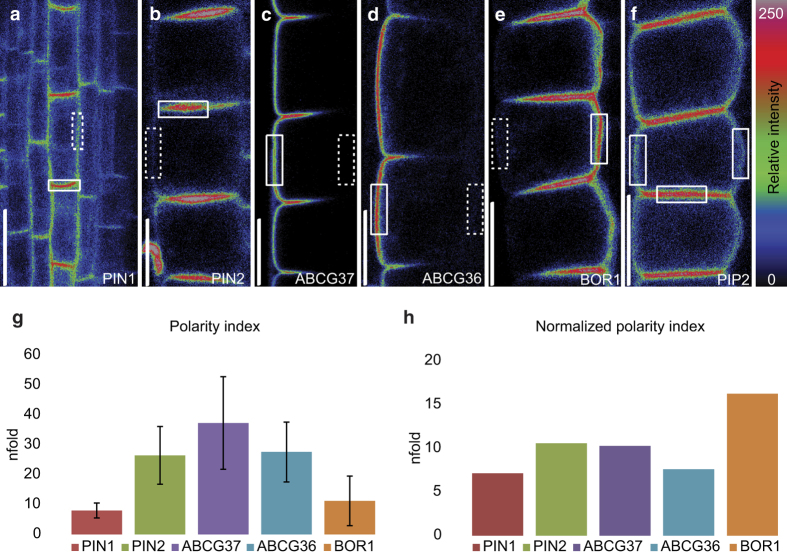
Polarization level of various PM cargos. (**a**–**f**) Relative fluorescence intensities showing predominantly a basal localization of PIN1-GFP in stele cells (**a**), an apical localization of PIN2-GFP in epidermal cells (**b**), an outer lateral localization of GFP-ABCG37 in epidermal cells (**c**), an outer lateral localization of ABCG36-GFP in epidermal cells **(d**), an inner-lateral localization of BOR1-GFP in epidermal cells (**e**), and an uneven distribution of PIP2-GFP (**f**). The relative fluorescence intensities are color coded from 0 (black) to 250 (bright/white). Scale bars=10 μm. (**g**) Quantification of the polarity index for steady-state PIN1-GFP, PIN2-GFP, GFP-ABCG37, ABCG36-GFP, and BOR1-GFP. The polarity index was calculated as the maximal signal ratio between the defining polar domain (full white rectangle) and it’s adjacent or opposite domain (dashed white rectangle). In the case of PIN1-GFP, the signal at the lateral domain was divided by 2 accounting for the equal contribution of fluorescent signal from two adjacent membranes. (**h**) Quantification of the normalized polarity index for steady-state PIN1-GFP, PIN2-GFP, GFP-ABCG37, ABCG36-GFP, and BOR1-GFP. Normalized index was generated by dividing the initial polarity index of each marker (**g**) by the corresponding PIP2-GFP polarity index (Supplementary Figure S1B). Error bars represent s.e.m.; *P*-value calculated according to Student’s *t*-test. *n*=80–100 cells from 20 to 25 roots.

To characterize systematically the different polar PM domains in plant cells, we evaluated quantitatively the subcellular localization of the green fluorescent protein (GFP)-fused polar cargos PIN1-GFP [42], PIN2-GFP [43], GFP-ABCG37 [44], ABCG36-GFP [45], and BOR1-GFP [40], and compared with the non-polar plasma membrane intrinsic protein 2A (PIP2-GFP; [46]) marker in roots of *Arabidopsis thaliana*. The stele-expressed PIN1-GFP showed predominantly basal signal enrichment with a weak lateral signal (Figure 1a). In the epidermis, PIN2-GFP had a very pronounced apical and a minor lateral signal that gradually decreased toward the bottom side of the cell (Figure 1b). GFP-ABCG37 and ABCG36-GFP localized largely to the outer domain of epidermal cells (Figure 1c and d) and BOR1-GFP to the inner PM domain (Figure 1e) with some enrichment at the apical and basal cell sides. Surprisingly, the presumed non-polar PIP2-GFP marker showed a not entirely symmetric signal between the polar domains, when imaged within the dynamic range of the photomultiplier (Figure 1f). The PIP2-GFP signal intensity was the strongest at the transversal (apical/basal) domains, less pronounced at the outer domain, and was weakest at the inner side of the cell (Supplementary Figure S1A–D). In an attempt to identify better, non-polar cargos than PIP2-GFP, we tested other presumed non-polar markers. Interestingly, detailed analysis of brassinosteroid-insensitive1 (BRI1)-GFP [47] and novel plant snare12 YFP [48] revealed signal distributions that were similar to that of PIP2-GFP, with strongest signal at the transversal and the weakest at the inner-lateral cell side (Supplementary Figure S1A and S1E–H). Moreover, all studied non-polar markers showed a peculiar signal distribution at the transversal domains, decreasing from the exterior toward the interior root end (Supplementary Figure S1). This suggests that perfect, non-polar markers probably do not exist in the context of a root meristem. Therefore, for further analyses we decided to use PIP2-GFP, the marker with the strongest signal intensity (which facilitates the fluorescence recovery after photobleaching (FRAP) analysis) and low signal intensity ratios between domains (~4/2/1—transversal/outer/inner). The transversal domain displayed the strongest signal compared with the outer domain, probably due to overlapping signals of abutting cell membranes. On the other hand, the inner membrane showed a much lower signal, which could reflect PIP2 protein function and tissue context (Supplementary Figure S1A). Altogether, PIP2-GFP showed a lower polarization level than any other tested polar marker.

For each cargo, we calculated a ‘polarity index’ defined as the ratio between the mean of the maximal signal intensity at a given polar domain and the least intensely labeled domain (lateral or opposite to polar; Figure 1a–f and Supplementary Figure S1B). Although the basally polarized PIN1-GFP showed the lowest polarity index, the actual distribution asymmetry must be much higher, since the PIN1-GFP signal intensity is gradually decreasing along the lateral domain presumably reaching its minimum at the opposite cell side, the apical domain that is masked by the signal derived from the basal domain of the above (more shootward) stele cell. However, because the lateral PIN1-GFP signal originates more or less equally from two adjacent membranes, we divided the measured lateral signal by 2 to calculate the PIN1-GFP polarity index (Figure 1g). Moreover, we aimed to take into account potential imaging artefacts derived from differences in cell shape and tissue thickness, as reflected in PIP2-GFP fluorescence variability at particular polar domains (Supplementary Figure S1A). Therefore, we normalized the polarity indices to the PIP2-GFP polarity index (Supplementary Figure S1B) providing more conservative estimates of the polarity indices (Figure 1h). Overall, all tested polar markers can be ordered as follows according to their polarity indices: BOR1>PIN2>ABCG37>ABCG36>PIN1>PIP2. In addition, we generated three-dimensional (3D) reconstructions (0.4 μm step) for each polar marker that contained preferential signal gradients in respective polar domains (Supplementary Figure S2A–E). Systematic evaluation of polar cargos under uniform conditions revealed different strengths of protein polarization, but all tested polar cargos showed clearly asymmetric localizations.

### Polarized endocytic recycling to the polar domain centers

**Figure 2:**
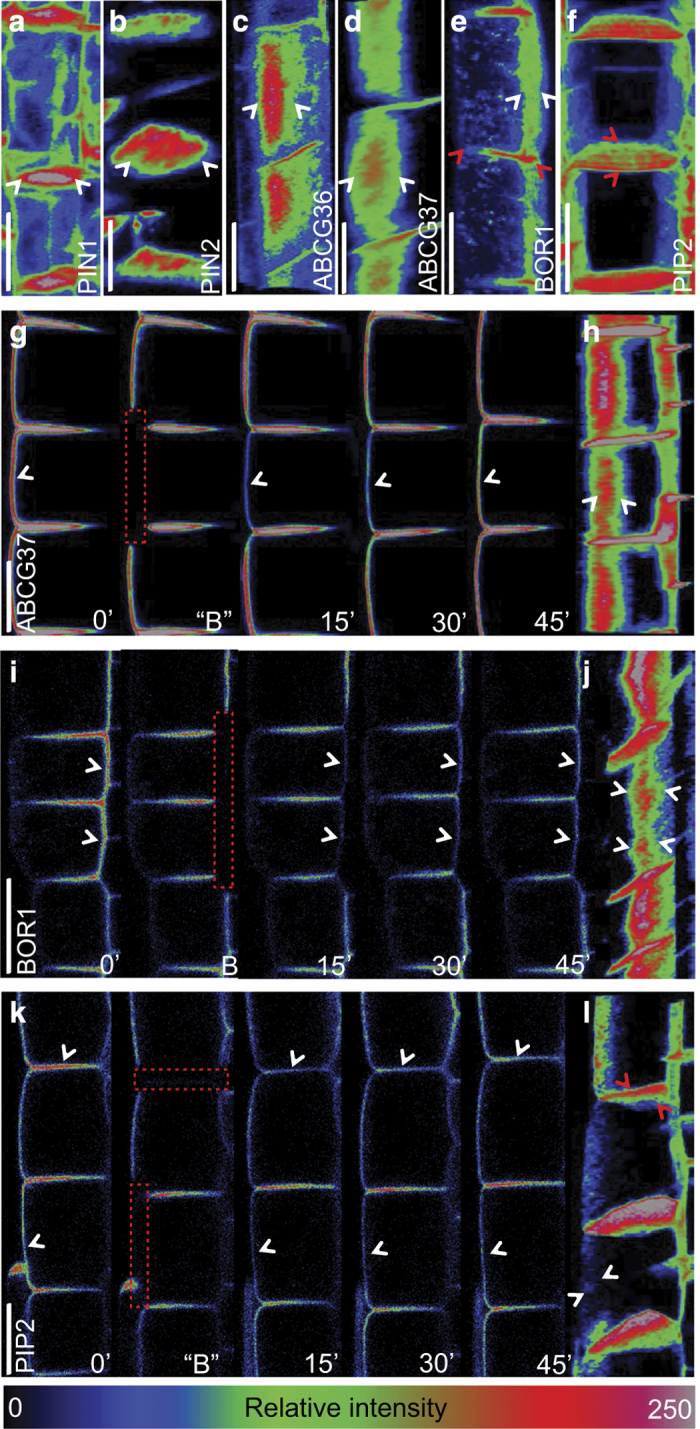
Super-polar delivery revealed by 3D reconstructions of polar cargo localizations. (**a**–**f**) Projected 3D reconstructions of z-stacks of different polar markers, highlighting their spatial distribution in the respective polar domains for PIN1-GFP (**a**), PIN2-GFP (**b**), ABCG36-GFP (**c**), GFP-ABCG37 (**d**), and BOR1-GFP (**e**). The white arrowheads mark the gradual signal decrease at the edges of respective polar domain. In addition, at transversal domains (merged apical/basal), BOR1-GFP and PIP2-GFP show a strong signal gradient decreasing toward the root surface (**e**) and from the root surface (**f**), respectively (red arrowheads). With the exception of PIN1, which occurs in the stele, all markers were analyzed in epidermal cells. (**g**–**l**) Dynamics of fluorescence recovery of GFP-ABCG37, BOR1-GFP, and PIP2-GFP over a time-course of 45 min after photobleaching (dashed red rectangles indicate photobleached regions). In single z-sections an equal signal recovery over the whole length of the domain (arrowheads) can be seen for GFP-ABCG37 (**g**), BOR1-GFP (**i**), and PIP2-GFP (**k**). In contrast, on 3D reconstructions at 45 min after photobleaching GFP-ABCG37 (**h**), BOR1-GFP (**j**), and PIP2-GFP (**l**), reveal a signal enrichment in the middle core of outer lateral domains (white arrowheads). In addition, non-polar marker PIP2-GFP shows different recovery pattern at transversal domain in comparison to outer lateral domain, displaying signal recovery at the periphery of the domain instead of in the middle core (**l**) (red arrowheads). Relative fluorescence intensity from 0 (black)–250 (bright/white) is represented by the color code. *n*=6–8 FRAP experiments on different roots. Scale bars=10 μm.

Previously, the constitutively endocytosed PIN2 had been shown to undergo polar recycling to the center of the apical PM domain [18]. We examined how the reporter signal was distributed at different polar domains for the different polar cargos and how these cargos were delivered to the respective target domains. For this purpose, we used 3D reconstructions (*x*, *y*, and *z*) of each reporter and implemented color-coded fluorescence intensity profiles to visualize steady-state protein levels semi-quantitatively. The 3D reconstructions were maximally projected along the angle that allows best visualization of the polar domain depending on the position of the domain. For example, in the case of PIN2, the 3D reconstruction was rotated to get a top view on the apical domain (Figure 2b), whereas for ABCG37, the reconstruction was rotated to get a side-view on the outer-lateral domain (Figure 2d). The PIN1-GFP signal was substantially higher in the center of the basal cell surface than that at the domain edges and at the lateral membrane domains (Figure 2a and Supplementary Figure S2A). Similarly, PIN2-GFP (Figure 2b and Supplementary Figure S2B), GFP-ABCG37, ABCG36-GFP, and BOR1-GFP showed strongest GFP fluorescence at the central zone of their respective polar domains (Figure 2c–e and Supplementary Figure S2C–E). On the other hand, the variable angle and tissue thickness may result in imaging artefacts. Therefore, we further tested the reliability of the observed protein enrichment by comparing the PM signal distribution of the endocytic tracer FM4-64. Owing to dye accessibility, the outer domain showed predominantly stronger staining than proximal and distal domains. Importantly, in contrast to GFP-tagged markers the signal did not show a graded distribution within any domain but remained equally distributed along the entire domain, suggesting that the observed signal intensity gradients are no imaging artefacts (Supplementary Figure S2G). The analysis of the top view of 3D reconstruction of transversal domain in stele and outer and inner-lateral domains in epidermis marked by PIN1-GFP, GFP-ABCG37 and, respectively, BOR1-GFP, did not show any signal drop-off in the *z*-dimension but a clear signal maximum of PIN1-GFP, GFP-ABCG37 in the central zone of their corresponding polar domains (Supplementary Figure S2F). Moreover, the analysis of BOR1-GFP confirmed its gradual signal distribution, from inner to outer domain, dropping along the proximal domain (Supplementary Figure S2F). Together, these data suggest that polar protein cargos are typically enriched in the central regions of their respective polar domains.

Previous observations suggested that the enrichment of PIN1 and PIN2 at the center of their domains in epidermal cells is due to the constitutive ‘super-polar’ recycling of cargos to these PM regions [18]. To test whether a similar mechanism also operates at lateral PM domains, we photobleached entire outer (GFP-ABCG37) and inner (BOR1-GFP) cell sides and subsequently followed the presumable recycling-based recovery within 15–45 min. During the FRAP in a single optical section, the signals of GFP-ABCG37 and BOR1-GFP were not visibly enhanced toward the center of the domain (Figure 2g and i). Only closer examination of the image z-stacks (0.4 μm steps) of all root epidermal cells after 45 min of bleaching revealed a signal intensity gradient from the center to the edges of the lateral domains with highest signal at the center (Figure 2h and j and Supplementary Figure S3A and B). Importantly, previously it has been demonstrated that PIN’s lateral mobility is similar between the middle core and periphery of a single-polar domain arguing against an important effect lateral diffusion and specific protein retention on polarization [18]. This suggests that the super-polar cargo delivery to the center of the respective polar domain also occurs for outer (GFP-ABCG37) and inner (BOR1-GFP) lateral cargos.

To test whether super-polar recycling to a particular polar domain is typically associated with polar cargos or applies to all PM proteins, we examined recycling of non-polar PIP2-GFP to outer and transversal cell sides. Similarly, 45 min after photobleaching of PIP2-GFP, at the outer domain there was no clear signal intensity gradient, but a somewhat dispersed signal with some preferential signal recovery at the center of the outer domain as observed by z-stack imaging (Figure 2l and Supplementary Figure S3D). In contrast, a similar analysis of the PIP2-GFP at the transversal domain revealed preferential recovery at the periphery of the polar domain resembling the steady-state situation (Figure 2f, k and l and Supplementary Figure S3C). These two distinct signal gradients at different polar domains (transversal– periphery, outer lateral—center) for a single cargo raise the question whether the same protein within the same cell can be delivered to different domains in a different manner. One can speculate that the delivery mechanism is the same, while endocytosis rates and regulation differs between the distinct domains. Alternatively, secreted proteins can be retained at the specific zones of the PM due to differential membrane composition. Although these two scenarios cannot be distinguished directly, we regularly observed that polar markers were delivered preferentially to the center of each polar domain and that their final distribution most likely depended on other factors such as protein mobility and stability within the specific regions of the PM. It also appears that super-polar recycling is not typical for polar proteins but other protein-specific delivery mechanisms, such as observed for the transversal domain of BOR1-GFP and PIP2-GFP exist and need further investigation.

### Lateral diffusion as a significant factor for polarity maintenance

Another relevant aspect of the polar cargos distribution could relate to their lateral diffusion within the PM, as was demonstrated for PIN cargos [18]. The kinetics of protein diffusion are mainly determined by membrane fluidity, binding kinetics of molecules to anchored or slowly moving structural components [49], and, additional interaction with the extracellular matrix (cell wall) [30].

**Figure 3:**
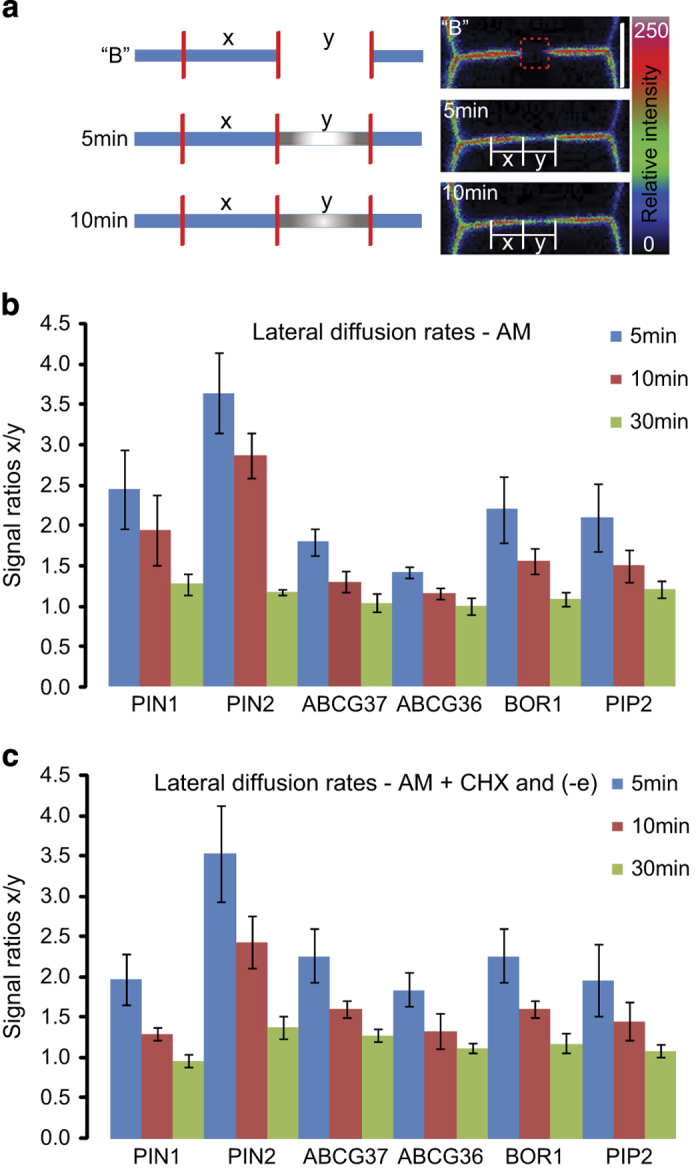
FRAP-based lateral diffusion measurements of cargos at apical, basal, outer, and inner domains. (**a**) Cartoon of the experimental set-up for estimating lateral diffusion indexes as defined by the evolution ratio of the average signal intensity of ‘x’ (nonbleached PM) over ‘y’ (bleached PM). Each region was 2 μm long. Relative fluorescence intensities from 0 (black) to 250 (bright/white) are represented by the color code. Scale bar=6 μm. (**b**, **c**) Evolution of signal ratio ‘x/y’ over 30 min for PIN1-GFP, PIN2-GFP, GFP-ABCG37, ABCG36-GFP, BOR1-GFP, and PIP2-GFP in control conditions (**b**) or under conditions where active processes were blocked (-e, 0.02% sodium azide and 50 mM 2-deoxy-D-glucose, and 50 μm cycloheximide; 45 min pretreatment) (**c**). The signal values of prebleach and postbleach fluorescence intensities were normalized and are s.e.m., *n*=4–5 FRAP experiments on different roots.

To obtain an insight into protein mobility in apical, basal, outer, and inner polar PM domains, we performed FRAP on a 2-μm subregion within the polar domain followed by semi-quantitative imaging of fluorescence recovery (Figure 3a and Supplementary Figure S4). Unlike previous studies [18, 50], which examined very short recovery times (up to 2 min), we performed long-term, diffusion-based recovery, addressing the total protein mobility. This type of experiment allows to capture the eventual motion of putative protein ‘clusters’ that in short-term, diffusion-based recovery experiments are perceived as non-mobile fraction. The recovery process was registered at three different time points, namely 5, 10, and 30 min. For all cargos, the fluorescence was restored to more or less prebleach levels within 30 min. However, after 5–10 min, the recovery of PIN2-GFP was weaker than that of other proteins (Figure 3b and Supplementary Figure S4) indicating a slower lateral diffusion of PIN2-GFP compared with other polar cargos. Fluorescence recovery can originate from several sources: lateral diffusion of proteins from neighboring PM regions, secretion of *de novo* synthesized proteins, or recycling of endocytosed proteins. To assess the contribution of the lateral mobility in the recovery process, we inhibited the ATP-dependent processes with sodium azide and 2-deoxy-D-glucose as well as protein biosynthesis with cycloheximide to exclude contributions of all active processes (Supplementary Figure S5A and B) [20, 49, 51]. This allowed us to focus specifically on the effect of passive, lateral diffusion on the fluorescence recovery (Figure 3c and Supplementary Figure S5C–H). After treatment with these inhibitors, the signal recovery pattern resembled that of untreated plants, suggesting that the impact of secretion and recycling on the signal recovery were marginal within 10 min after photobleaching (Figure 3c). The differences in fluorescence recovery between PIN2-GFP and other polar cargoes, such as PIN1-GFP or GFP-ABCG37 were very clear, hinting at a relatively lower lateral diffusion rate of the PIN2-GFP protein. Consistently with these findings, the lateral diffusion rates of PIN2-GFP and ABCG37-GFP extracted from FRAP imaging data as described [52] were 0.000138±0.0000285 μm^2 ^s^−1^ and 0.145±0.0597 μm^2^ s^−1^, respectively (Supplementary Figure S6). Despite that our results represent only relative differences and estimates, it remains clear that the lateral diffusion rates of the polar proteins, with exception of PIN2, were similar to those of PIP2-GFP, suggesting that a limited lateral diffusion is unlikely to be a unique property of polar cargos, implying the involvement of additional mechanisms to maintain a polar distribution of polar cargos.

### Lateral diffusion rates do not depend on polar domain or cell type

Given the negligible contribution of secretion and polar recycling in the 10-min time window of FRAP (Figure 3 and Supplementary Figures S4 and S5), the observed differences in lateral diffusion rates between different cargos might be due to alternative PM compositions of the different polar PM domains thereby altering the retention ability. To test this hypothesis, we evaluated the PIP2 lateral mobility in epidermal cells of transversal and outer domains (Supplementary Figure S7A). In the first 10 min of experiment the difference in protein mobility between distinct domains was significant, suggesting that PM composition may have an impact on PIP2-GFP signal intensity at different domains as well as distribution within single domain.

To test lateral diffusion rates of polar cargos in different cell types, we analyzed the mobility of the ectopically expressed PIN1-GFP in the epidermis (PIN2::PIN1-GFP2) and within its endogenous expression domain in the stele (PIN1::PIN1-GFP; Supplementary Figure S7B). No significant differences in the PIN1-GFP lateral diffusion could be observed in these different expression domains. Therefore, lateral diffusion of PM proteins may not depend so strictly on a particular cell type, but rather on the identity and protein sequence of each individual cargo, as has recently been suggested via single-particle tracking PALM analyses of different membrane proteins [53].

### Polar cargo clustering at different polar domains

**Figure 4:**
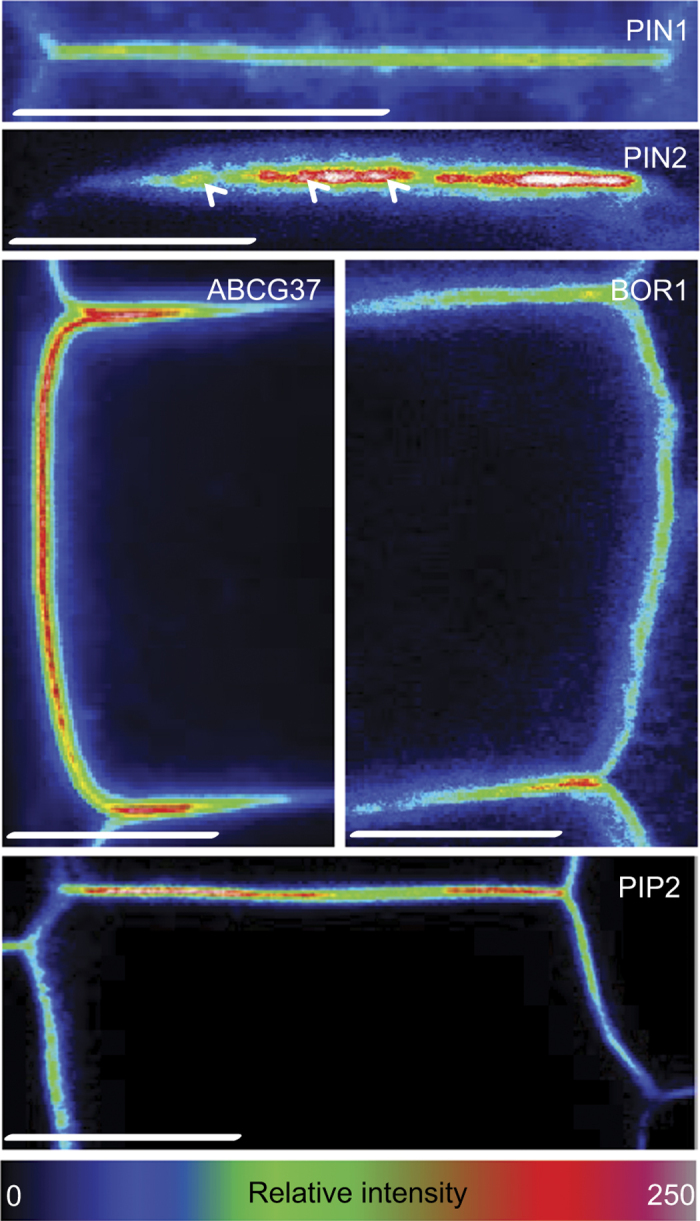
Clustering of cargos at the polar domains. Live imaging on PIN1-GFP expressed in stele, PIN2-GFP, GFP-ABCG37, BOR1-GFP, and PIP2-GFP in root epidermal cells. The arrowheads mark PIN2-GFP signal heterogeneity in the PM, or so-called ‘clusters’, which were not apparent for the other PM cargoes. Fluorescence intensity from 0 (black) to 250 (bright/white) is represented by the color code. This result was observed at least three times for each marker *n*=100–120 cells on 10–12 different roots. Scale bars=5 μm.

Protein lateral diffusion within the PM depends on the protein ability to interact with other PM components and on their aggregation with the PM [49]. Previously, the relatively low lateral diffusion rates of PIN1 and PIN2 have been suggested to be related to their uneven, more discrete distribution at the polar domains in so-called ‘clusters’ [18]. So far, these clusters have been observed with PIN2-GFP and the ectopically expressed PIN1-GFP at the apical and basal domains in epidermal cells. To assess whether cargo clustering mechanism could be a common phenomenon of different polar cargos or, alternatively, a specific feature of individual proteins, we tested proteins that localized at lateral domains, such as GFP-ABCG37, BOR1-GFP, and, as a reference, PIN2-GFP and PIP2-GFP in the epidermis and PIN1-GFP in the stele (Figure 4). Although signal heterogeneity and protein clusters were visible on live imaging for PIN2-GFP, we were unable to see comparably strong clustering for any of the other cargos (Figure 4), suggesting that clusters might be formed only in certain domains or be a protein-specific feature. However, we could not observe such signal heterogeneity for PIP2-GFP in any polar domain. This suggests that clustering or confinement behavior most likely depended on specific properties of the individual proteins. Another possible explanation for the lack of protein clusters in the live-imaging studies of other polar cargos might be due to the limitations of confocal microscopes to detect tiny and densely packed aggregates combined with a higher proportion of freely diffusing proteins. To expose putative weaker or smaller and more frequent agglomerations, we fixed the seedlings and treated all the samples according to the immunostaining protocol [54]. As the fused-GFP proteins were well preserved we did not need to use anti-GFP antibodies to visualize the proteins. The immunostaining protocol improved the visualization of the signal heterogeneity throughout all the polar PM domains of the analyzed markers but still retained the relative differences in the clustering of different cargoes (Supplementary Figures S8 and S9). These findings imply that at least some protein clustering in the PM domains might be a common feature of plant PM proteins, whereas the high clustering degree is most likely a specific attribute typical for some polar protein cargos such as PIN2.

Although no direct relationship between membrane sub-compartmentalization and lateral mobility has been clearly established [30], PIN2-GFP displayed the most pronounced clusters correlating with a very low lateral diffusion rate of PIN2 and the most pronounced polarity of its distribution. In addition, inhibition of clustering by filipin-mediated sterol depletion gave rise to a higher PIN2-GFP diffusion rates [50]. Given these correlations, one can speculate that the cargo clustering in the polar PM domains contributes to limiting their lateral diffusion within the PM.

### Cell wall importance for polarity maintenance

**Figure 5:**
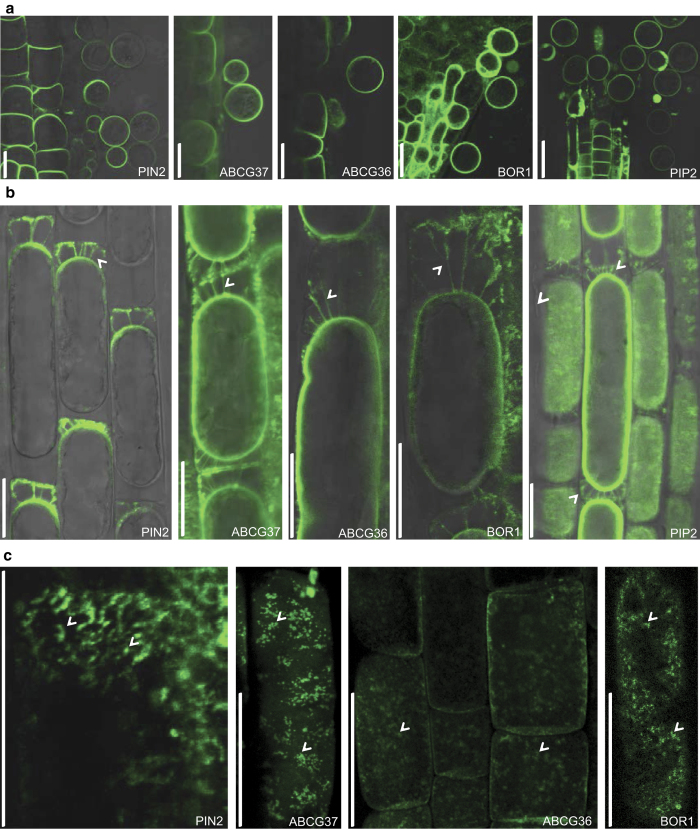
Maintenance of cargo polar distributions by connections between PM and cell walls. (**a**) On cell wall digestion (cellulase+macerozyme), the resulting protoplasts showed an immediate polarity loss for all tested GFP-fused markers. (**b**) Plasmolysis (mannitol+macerozyme) revealed that all tested GFP-fused PM markers are connected to the cell walls by Hectian strands (arrowheads). Note a more pronounced protein association to the cell wall vs PM for PIN2-GFP compared to the other markers. (**c**) Projected 3D reconstruction after cell plasmolysis. Arrowheads depict protein ‘rafts’ anchored at the cell wall (upper view on apical domain for PIN2-GFP and lateral view on GFP-ABCG37, ABCG36, and BOR1-GFP in epidermal cells, respectively). Plasmolysis and protoplasting experiments were done at least three times for each marker (20–22 roots analyzed). Scale bar=10 μm.

The cell wall has been proposed to be an important factor for the maintenance of the polar cargo distribution at the PM [29]. To assess the role of the cell wall as a general component not only for apical and basal, but also inner and outer domains, we used GFP-fused cargos for different polar domains and non-polar PIP2-GFP and removed cell walls by protoplasting. The polar distribution of all tested proteins within the PM was rapidly lost and the originally polarized proteins became uniformly distributed at the PM (Figure 5a). These results demonstrate that the cell wall is important for polarity maintenance at all polar domains, probably assisting in restricting lateral diffusion [30].

Apical PIN2-GFP and basal PIN1-GFP have been shown to be enriched at the connections between cell wall and PM, the so-called Hechtian strands, visualized by mannitol-induced plasmolysis [29]. When we performed a similar experiment with other polar markers, Hechtian strands could be observed as early as after 20 min of partial degradation of the cell wall and plasmolysis (Figure 5b and c). Moreover, the initially somewhat asymmetric PIP2-GFP became uniformly distributed after plasmolysis underscoring the importance of the cell wall in differential protein accumulation.

Interestingly, all the polar and non-polar marker lines showed fluorescent signals at the PM-cell wall contacts at Hechtian strands (Figure 5b), suggesting that association with the cell wall is a mechanism not only reserved for polarly localized proteins but seems to be a common future of PM-localized proteins. The role of such association is still not fully understood; however, one possible explanation is a regulation of protein mobility within the PM. Inhibition of clustering by filipin-mediated sterol depletion [18] or cell wall digestion [29] results in an increases PIN2-GFP mobility, supporting this hypothesis. Overall, our data indicate that the cell wall integrity could be potentially important for the polarity maintenance at all polar PM domains. Therefore, the future challenge will be to determine the precise relationship between protein clustering, the cell wall and protein lateral diffusion.

### Polarized secretion contributes to polar cargo distribution

**Figure 6:**
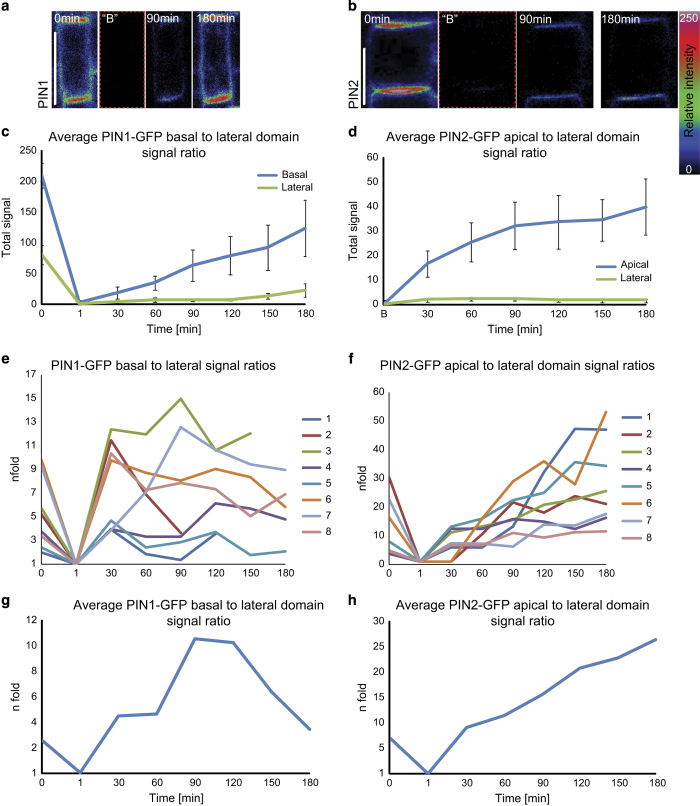
Quantitative analysis of PIN1 and PIN2 signal recovery at the PM after complete cell photobleaching. (**a**, **b**) Whole-cell FRAP of PIN1-GFP (**a**) and PIN2-GFP (**b**) shows predominant signal recovery at the respective polar domains. Relative fluorescence intensity from 0 (black) to 250 (bright/white) is represented by the color code. Scale bar=10 μm. (**c**, **d**) Quantification of the signal intensity recovery for PIN1-GFP (**c**) and PIN2-GFP (**d**) at the polar and non-polar domains. The signal values of prebleach and postbleach fluorescence intensities were normalized and error bars are s.e.m. *n*=8 FRAP experiments on different roots. (**e**–**h**) Dynamics of polarization of recovering fluorescent signal as depicted as the evolution of the ratio of signal intensities (polar vs lateral) for PIN1-GFP (**e**, **g**) and PIN2-GFP (**f**, **h**) over time. In the graphs **e**, **f** each of colored profiles (numbered 1–8) represent the ratio dynamics of signal intensities calculated from individual FRAP experiments.

As a limited lateral diffusion and polar recycling to the center of all polar domains might represent common mechanisms dictating the asymmetric distribution of different plant polar cargos, we investigated whether a *de novo* secretion of freshly synthesized proteins could contribute to the polar distribution. To address this issue, we photobleached all GFP-tagged cargos from a group of cells, acquired spatial fluorescence recovery profiles, and calculated the corresponding polarity indexes that reflect the spatio-temporal kinetics of the *de novo* synthesized and secreted polar cargos (Figure 6a and b and Supplementary Figures S10, S11, S12).

Half an hour after complete cell photobleaching, first measurable and strongest fluorescent signals were observed at respective polar PM domains suggesting a preferential polar cargo delivery (Figure 6c and d and Supplementary Figures S10, S11, S12, 13). During the progressing recovery, the signal intensities within the corresponding polar and non-polar domains increased. In most cases as exemplified by PIN1-GFP, GFP-ABCG37, ABCG36-GFP, and BOR1-GFP, the corresponding signal ratios revealed dynamic polarity index profiles that reached a transient signal peak before returning to a balanced steady-state level (Figure 6e and g and Supplementary Figures S11E–H, S12C and S13D), PIN2-GFP diverged from this pattern with a persistently increasing polarity index (Figure 6f and h and Supplementary Figure S10C). This may suggest that during 3 h of PIN2-GFP recovery we were able to capture only the initial phase (of polar index ‘growth’), which occurs for other markers within ~90 min after photobleaching. This is in line with a much lower recovery rate of PIN2-GFP (17%) in comparison to the others PM proteins, including PIN1-GFP (77%), within 3 h of recovery (Supplementary Figure S12F). Alternatively this result could be hinting at distinct mechanisms of polarity establishment and the presence of additional factors that modulate cargo trafficking and distribution. Together, these data suggest that besides a super-polar recycling, also a polar secretion of *de novo* synthesized proteins is part of a common mechanism of cargo delivery to distinct polar domains.

### Computer simulations of polarity generation and maintenance mechanisms

**Figure 7:**
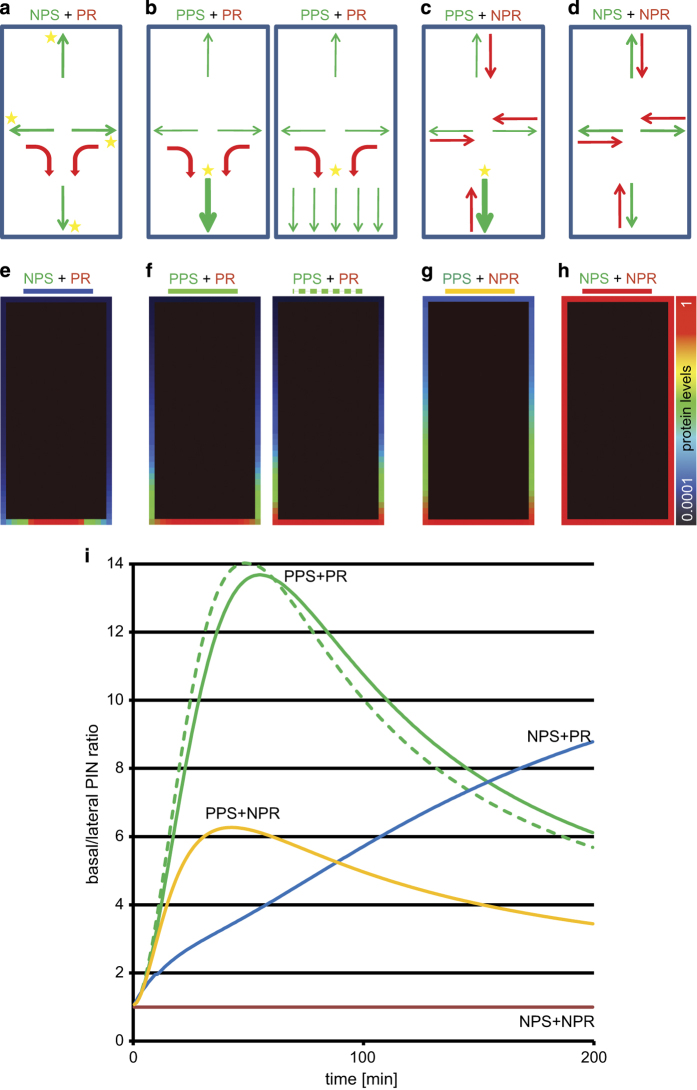
Computer simulations of two alternative secretion scenarios. (**a**–**d**) Hypothetical models assuming non-polar secretion and polar recycling (**a**); preferential polar secretion and polar recycling (left and right panels refer to protein polar recycling to the center and to the whole-polar domain, respectively) (**b**); preferential polar secretion and non-polar recycling (**c**); non-polar secretion and non-polar recycling model (**d**); (NPR, non-polar recycling; NPS, non-polar secretion; PPS, preferentially polar secretion; PR, polar recycling). The thickness of the arrows indicates the protein trafficking intensity (green, secretion; red, recycling), and the stars position the cargo-targeting specifying determinants. (**e**–**h**) Computer simulations of protein polarization referring to the hypothetical models as described above, respectively. Only model assuming non-polar secretion and non-polar recycling is completely unable to establish cell polarization (**h**). Protein levels are represented by color coding scheme, from low (0.0001) to high (1) (log scale). (**i**) Evolution of signal intensity profiles obtained after an extended simulation time for models: assuming non-polar secretions and polar recycling (blue line), polar secretions and polar recycling to the polar domain center (green solid line), polar secretion and polar recycling equally to the whole-polar domain (green dashed line), and as a reference models assuming polar secretion and non-polar recycling (yellow line), and non-polar recycling and non-polar secretion (red solid line). See also Materials and Methods section for a detailed description of the models.

Given the apparent multitude of processes involved in polarity establishment and maintenance in plant cells, it remains difficult to experimentally study the contribution of each of these processes to the cell polarization. Therefore, we performed an *in silico* dissection of the individual contribution of lateral diffusion, secretion, polar recycling, and protein clustering to the polarity dynamics at the single-cell level using an extension of a recently proposed computer model [18, 55]. We tested two possible hypotheses for the polarity generation and maintenance of polar PM domains in plant cells. The ‘non-polar secretion’ model (Figure 7a), integrating the assumptions that newly synthesized proteins were ubiquitously secreted to the PM in a non-polar fashion and subsequently polarly recycled between different cell sides based on sequence-specific modification signals (that is, protein phosphorylation; see Supplementary Methods). The non-polar secretion has been proposed previously [56], however, some of the key experiments were methodically questioned and we could not reproduce them using more advanced FRAP analysis also including 2-photon and spinning disc microscopy (Supplementary Figure S13). In contrast, in the ‘polar secretion’ model (Figure 7b), we assumed that *de novo* synthesized and recycled proteins were sorted and delivered to the PM in a polar fashion based on preexisting polarity cues. In both models, we used experimental estimates for lateral diffusion and secretion rates of representative polar cargos (GFP-ABCG37 and PIN2-GFP; Supplementary Figures S6 and S12). Besides lateral diffusion and protein synthesis, our computer model integrated previously estimated rates of endocytosis, degradation, and recycling described by coupled mathematical terms (for a detailed description of the models, we refer to the Methods section and our previous study [55]). At the onset of each simulation, the model represented a photobleached cell. Importantly, both simulations of hypothesized scenarios demonstrated that models with either non-polar secretion (Figure 7a and e) or preferential polar secretion (Figure 7b–d,f and g) were capable of generating a steady-state protein polarization, unlike the non-polar reference model that lacked polar secretion and polar recycling (Figure 7d and h).

To test whether these two models could reproduce the dynamic profile of the polarity index observed in microscopic studies of the PIN1, ABCG37, ABCG36, and BOR1 markers, we calculated the corresponding polarity indexes obtained with model simulations and plotted them as a function of time. The ‘non-polar secretion’ model predicted a monotonic increase of the polarity index in time (Figure 7a,e and i blue line), whereas the ‘polar secretion’ model (Figure 7b,f and i green line) displayed a dynamic profile of the polarity index changes that were characterized by strong pulse and a further diffusion-dependent stabilization at the steady-state level. Although the ‘polar secretion’ model predicted kinetics of the polarity index (Figure 7b and i, green line) that closely resembled that observed in the microscopic experiments for PIN1, ABCG37, and BOR1 (Figure 6e and g and Supplementary Figures S11E,G, S12C,D and S13D), the ‘non-polar secretion’ (Figure 7a and i, blue line) model could not reproduce *in vivo* observed polarity index changes attributed to PIN1, ABCG37, ABCG36, and BOR1 cargos for any set of given parameters (Supplementary Figure S14).

**Figure 8:**
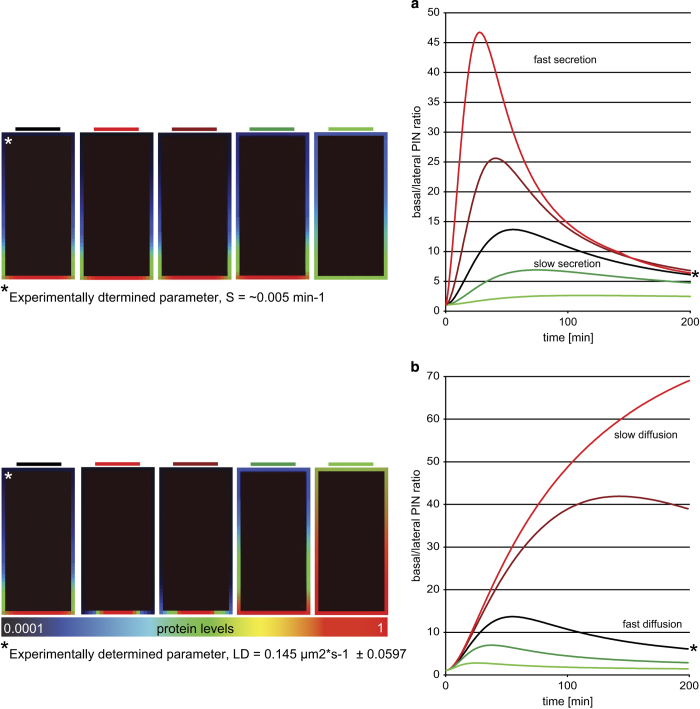
Strong impact of polar secretion and lateral diffusion parameters on polarity establishment and maintenance in the polar secretion model. (**a**) The polarity index profile dramatically changed depending on the secretion intensity. The model depicted by asterisk includes the experimentally obtained secretion parameter (based on the calculated half-recovery time of GFP-ABCG37 kSPEX ~0.005 min^−1^). Other models represent cell polarity variants dependent on secretion rate, from very high (red, kSPEX=0.5 min^−1^) to very low (light green, kSPEX=0.00005 min^−1^), accordingly. (**b**) Impact of lateral diffusion on the protein polarization dynamics. The model depicted by asterisks includes representative lateral diffusion parameters obtained experimentally efficiency for GFP-ABCG37 (0.145 μm^2^ s^−1^±0.0597). Other models represent cell polarity variants dependent on lateral diffusion, from very slow (red, Dm=0.00001 μm^2^ s^−1^) to very fast (light green, Dm=1 μm^2^ s^−1^), accordingly. Protein levels are represented by color coding scheme, from low (0.0001) to high (1; log scale).

Our simulations revealed that higher rates of protein secretion (Figure 8a) lead to increased height, reduced width, and reduced timing of this cargo-specific polarity index pulse that could possibly explain observed peak differences between different polar cargoes. Moreover, a decrease in lateral diffusion in the ‘polar secretion’ model eventually resulted in elimination of the pulsed dynamics and a preferentially monotonic polarity index profile (Figure 8b) that is reminiscent of that observed for the slowly diffusing and secreted PIN2 protein (F and H). Finally, the ‘polar secretion’ model predicted a similar tendency in polarity index change to that of reduced lateral diffusion (Figure 8b) and after imposing a strong degree of protein clustering in the PM domains (Supplementary Figure S15). Importantly, both predicted features are typical characteristics of the PIN2 protein (Figure 4a) [18].

These results indicate that the ‘polar secretion’ model provides a consistent and plausible explanation for the different mechanisms, including secretion, lateral diffusion, and clustering that contribute to the generation of polar cargo distribution at various polar domains of the plant cell. Moreover, our model simulations revealed that polar secretion rate and reduced lateral diffusion are important determinants of the polar cargo localization.

## Discussion

Polarity is fundamental for biological processes in both mammals and plants [4, 57–59]. Although the mechanism of how cells break symmetry is still not completely clear, a number of reports suggest that polarity can be determined based on various external or internal cues [4, 45]. These signals perceived by randomly localized receptors may recruit *de novo* synthesized or recycled effector proteins, which further initiate cytoskeleton and trafficking reorganization that establish a polar domain at the PM. Conceptually, the asymmetric distribution of polar cargos at the PM results from the combined secretion, endocytosis, and recycling back to the PM as well as from mechanisms limiting lateral diffusion. Here, we examined the contributions of these processes to the polar distribution of cargos at the apical, basal, outer, and inner polar domains and found a number of shared cellular processes that underlie the polar cargo distribution in all these polar domains.

### Polar secretion as an unappreciated process in polar cargo distribution

In plants, the process of polar secretion is not very well documented, possibly due to the difficulty to distinguish mechanisms guiding secretion from recycling back to the PM after endocytosis. For instance, several components required for the delivery of PIN proteins to the PM have been identified, such as the ARF GEF GNOM [21, 60] or the small GTPase BEX5/RabA1b [61], but their relative contributions to recycling of existing PIN proteins and secretion of *de novo* synthesized ones are unclear. Examples for specific, directional secretion include delivery of the syntaxin KNOLLE/SYP111 [62], and multiple other membrane cargoes [26] to the forming cell plate, polar secretion for the tip growth of pollen tubes or root hairs [63], or polar localization of phosphate transporter 4 in *Medicago truncatula* mediated by transient secretion reorientation [64]. However, the role of polar secretion in polarity has been underestimated, in part due to earlier controversial/questioned observations suggesting a non-polar secretion of *de novo* synthesized PIN proteins [65]. These observations were based on the FRAP experiments with an apparent non-polar recovery of PIN-GFP signal after complete photobleaching and non-polar PIN PM signal after strong induction of PIN overexpression [56, 65]. With more advanced FRAP analysis, microscopy and better signal quantification, we could not confirm the reported initial non-polar recovery of PIN-GFP PM signal. Instead, we observed polar cargo recovery at given polar domain, which certainly originates from *de novo* protein secretion but also could be amplified by immediate polar recycling. As these two processes cannot be clearly dissected and uncoupled we developed a computational model testing various scenarios.

Our experimental data in conjunction with the computational modeling favors the scenario that the polar proteins are initially delivered asymmetrically to the corresponding polar domains. However, because, according to the FRAP analysis, it takes an extended time to replace the initial pool of proteins, secretion alone cannot overcome the relatively fast process of lateral diffusion and maintain the polar distribution. This observation implies the existence of important additional mechanisms, such as constitutive endocytic recycling that would be able generating a polar distribution also from an originally symmetric situation. The importance of endocytic processes for polar PIN distribution has been extensively demonstrated by non-polar PIN distribution in mutants with defective endocytosis [19, 20, 23, 24, 65, 66]. Therefore, in light of our current observations, we propose that polar secretion of *de novo* synthesized proteins is a common process, which occurs to a different extend in different cell types. Simultaneously we highlight the essential role of constitutive endocytosis and recycling in polarity maintenance.

### Polar endocytic recycling as common process at all polar domains

Following the initial observation that PIN proteins undergo constitutive cycles of endocytosis and recycling back to the polar PM domain [17], it has been shown that dynamic endomembrane trafficking is crucial for polar cargo distribution [23, 65, 67]. Our results suggest that the recycling to all polar domains is highly polar and constantly delivers cargos to the center of the polar domain where also the highest concentration of the cargos is detected. In addition, internalization of polar cargos, such as the PIN proteins, can be inhibited specifically at the polar domains by negative regulators of endocytosis, the MACCHI-BOU (MAB) proteins [68]. These mechanisms not only constantly reinforce and maintain the seemingly static polar cargo distribution in polarized cells, but also presumably allow rapid polarity changes in different developmental processes, such as embryogenesis [69], organogenesis [42, 70], vascular tissue formation [71], and regeneration [72], fruit development [73], or in response to different external cues, such as light, gravity, or pathogen infection [45, 74–76].

Another potential role for the endocytic recycling, besides redirecting cargos between different polar domains, might be the regulation of the amount and, thus, the activity of proteins at the PM, hence, providing a possibility to redirect the cargo traffic to the vacuole for degradation. The decision between recycling and vacuolar targeting also seems to be influenced by different signals, including signaling molecules [77–80] as well as nutrients, such as boron [41]. It would be interesting to gain further insights into how particular lateral cargos, such as those related to the exchange of substances between plant and environment, are regulated at the level of the constitutive endocytic recycling.

### Common mechanisms limiting lateral diffusion at the polar domains

In addition to secretion and endocytic (re)cycling, polar cargo distribution has been shown to involve mechanisms limiting the cargo confinement of polar cargos within the fluid environment of the PM, such as cargo clustering, the extracellular matrix, the actin cytoskeleton, and the plant cell walls [18, 29, 30]. Our observations of cargos at different polar domains revealed that independently of polar domains and cargos, the polar cargos show not only an inhibited lateral diffusion, but also various degrees of clustering. PIN2 at the apical domain possesses the slowest lateral diffusion, implying that clustering indeed limits lateral diffusion. However, at the moment, the data are too limited to understand the mechanistic connection of these two phenomena. In contrast, the extracellular matrix, the cell wall, and its connection to the PM [29, 30] seem to be common for all polar domains, because removal of the cell wall has a pronounced impact on the polar distribution of all cargos tested. Examination of the nature and exact role of the junctions between polar domains and cell walls as well as understanding the correlation between clustering of polar cargos and their lateral diffusion will be the major challenge in the future years. In addition, the implementation of single-particle tracking and other more advanced imaging techniques on polar cargoes will allow further dissecting cargo confinement mechanisms that control lateral diffusion.

## Materials and methods

### Plant material and growth conditions

The transgenic lines PIN1::PIN1:GFP [42], PIN2::PIN1:GFP [81], 35S::GFP:PIS1/35S::GFP-ABCG37 [44], PEN3::PEN3:GFP/ABCG36::ABCG36-GFP [82], BOR1::BOR1:GFP [41], and 35S::PIP2:GFP [46], PIN2::PIN1:GFP-2, PIN2::PIN1:GFP-3, eir1-1 [33], BRI1::BRI1:GFP [47], and UBQ10::YFP-novel plant snare12 [48] have been described before. Seeds of *Arabidopsis thaliana* (L.) Heyhn. were sterilized with chlorine gas and stratified at 4 °C for 2 days in the dark. Five-day-old seedlings were grown on vertically oriented plates containing *Arabidopsis* medium consisting of half-strength Murashige and Skoog medium supplemented with 0.8% agar, and 1% sucrose (pH 5.9) under a 16-h/8-h photoperiod at 22°/18 °C. For the FRAP analysis, plants were grown vertically on the plate for 5–6 days, then placed on a chambered cover glass (Nunc Lab-Tek), covered with a slice of *Arabidopsis* medium medium, and scanned as indicated. Because of the degradation of BOR1 under high boron conditions, the BOR1-GFP line was tested with a special boron-deficient medium containing 0.3 μM boric acid [41].

### Drug treatments

To assess the lateral diffusion rate, first we checked the energy inhibitor efficiency. The endocytosis rate of treated and nontreated seedlings was tested with the endocytic tracer *N*-(3-triethylammoniumpropyl)-4-(6-(4-(diethylamino) phenyl) hexatrienyl) pyridinium dibromide (FM4-64) (Molecular Probes). The control seedlings were incubated for 10 min in the presence of 4 μM FM4-64, washed out, and checked with a confocal laser scanning microscope (Zeiss 710). Seedlings treated with inhibitors, where initially pretreated with 50 μM cycloheximide (Sigma-Aldrich) and with energy inhibitors (-e, 0.02% sodium azide, and 50 mM 2-deoxy-D-glucose) [20] for 35 min and with cycloheximide, -e, and 4 μM FM4-64 for 10 min. All treatments were carried out in sterilized liquid *Arabidopsis* medium (no agar) at room temperature in the light and at least in triplicate with a minimum of 12 roots for each treatment, unless stated otherwise. Only one treatment with FM4-64 was done on ice. Control treatments contained an equal amount of solvent (dimethylsulfoxide).

### Cluster visualization

*Arabidopsis* seedlings were fixed in 4% paraformaldehyde in phosphate-buffered saline (PBS) for 1 h. Samples were washed with PBS/0.1% Triton (5×10 min) and water (5×10 min). Cell walls were partially digested with 2% driselase (Sigma-Aldrich, Hamburg, Germany) in PBS for 45 min and subsequently washed with PBS/0.1% Triton (5×10 min). Samples were incubated in 10% dimethylsulfoxide and 3% NP-40 in PBS for 1 h. After extensive washing with PBS/0.1% Triton (8×10 min), samples were washed with water (5×10 min).

### Polarity quantification

The mean fluorescence signal intensity of different GFP-fused lines at the polar and opposite or lateral sides of cells (as indicated in Figure 1) were measured with ImageJ 1.40 g (http://rsb.info.nih.gov/ij/). This software allows the drawing of lines of the same length along each of analyzed cell sides. The obtained mean pixel intensity values of certain lengths were then used to generate recovery curves and determine the polarity index—the ratio of X protein intensity at the polar vs the lateral or opposite sides. Polarity index of PIN1-GFP was further modified. Because, the measured signal at the lateral domain originates assumingly equally from two neighboring cells, all the obtained values were divided by 2 resulting in twofold increase of polarity index. Similar rule was not applied to other markers due to differential expression in epidermis and cortex as well as the fact that signal measured at outer lateral domain originates from a single membrane. Taking into account the signal interference from differential tissue thickness and cell shape, which results in imperfect polarity index between polar domains (Supplementary Figure S1A), obtained polarity indexes (Figure 1g) were further normalized to/ divided by corresponding PIP2-GFP polarity index (Supplementary Figure S1B).

### Microscopy

For the confocal laser scanning microscopy, we used a Zeiss 710 or Olympus fluoview FV10 with an inverted microscope setting. Semi-quantitative confocal imaging was analyzed with the Zeiss 710 microscope. Images were processed in Adobe Photoshop CS10 and assembled in Adobe Illustrator CS10 (Adobe Inc., London, UK). The fluorescence signal intensity was analyzed with ImageJ 1.40 g (http://rsb.info.nih.gov/ij/) and the provided confocal software (Zeiss and Olympus). The data were statistically evaluated with Excel 2007 (Microsoft). All the 3D reconstructions were done with the Zeiss 710 microscope at a 0.4–0.5 μm interval size.
