# Supplementary material for: Cellular mechanisms for cargo delivery and polarity maintenance at different polar domains in plant cells
Source: Cell Discov. 2016 Jul 19;2:16018–. doi: 10.1038/celldisc.2016.18 (PMC4950145; doi:10.1038/celldisc.2016.18)
Supplement: Supplementary Information [file celldisc201618-s1.pdf]

Supplementary Methods:

Protein Superpolar Recyling - FRAP on Entire Polar Domains

The entire polar domains were bleached 2x at 100% laser power and with 100 iterations. The bleaching stopped when the signal dropped below 1% of the initial intensity. Postbleach scans were done at 3% laser transmission every 15 min until 45 min. The GFP emission was detected between 505 and 530 nm. To test whether the localized recovery indeed occurred, 3D reconstructions of bleached cells were made at 0.5-µm interval size.

Protein Mobility - FRAP on 2 µm-Wide Regions of Polar Domains

Two-µm PM regions were bleached at 100% laser power and with 80 iterations. The bleaching stopped when the signal dropped below 1% of the initial intensity. First, the signal intensities at the prebleached region (ROI) and congruent (neighboring) region on the same domain were measured using ImageJ software (http://rsb.info.nih.gov/ij/). Next, the 2-µm ROI were bleached and subsequently the signal intensity at the ROI and congruent region was measured. In total, four time points: 0 min, "B", 5 min, and 10 min after bleaching were registered. Postbleach scans were done at 3% laser transmission and the GFP emission was detected between 505 and 530 nm. For analysis of the FRAP data to assess the protein lateral diffusion rate and generate the ratios we normalized the intensities with the equation: *In=(It1)/(It2-Imin2)*; where *In* is the normalized intensity; *It1*, the mean intensity at any time on the nonbleached neighboring PM region (2 µm) "x" (see Figure 3); *It2*, the mean intensity on the bleached ROI (2 µm) "y" at any time after postphotobleaching; and *Imin2*, the minimum postphotobleaching intensity on the bleached ROI "y". Because, all tested markers, except PIN2-GFP, showed comparably high lateral diffusion rates, parameters obtained for GFP-ABCG37 were considered as representative. Diffusion rates of PIN2 (0.000138 $\mu m^2 * s^{-1}$ ± 0.0000285) and ABCG37 (0.145 $\mu m^2 * s^{-1}$ ± 0.0597) were estimated with previously proposed methods for FRAP image analysis (Goehring et al., 2010). These diffusion rates were used in computer model simulations as default/nominal values. In order to avoid the focus drift all the pictures were taken manually in regular time laps instead automatically. Additionally all the pictures for all the markers were taken in the same way, therefore the eventual drift applies to all the markers and results equally.

Protein Secretion - FRAP on Entire Cell

For the FRAP analysis, we used an inverted Zeiss 710 microscope with a water-immersed 40× objective and a 488-nm diode laser excitation. The GFP emission was detected between 505 and

530 nm. The cell of interest as well as the neighboring cells were bleached to avoid the influence of lateral diffusion from neighboring cells. Depending on the signal intensity of the line, different bleaching settings were used. All the cells were bleached twice using following settings: 100% laser power until the initial signal intensity in ROI dropped below 1%; the iterations number was 100 for all the markers localizing in the epidermis and 200 for PIN1 in the stele; postbleach scans were done at 3% laser transmission and taken every 30 min until 180 min. For PIN1, additional scans were done at the early recovery stages as indicated (Figure 3). The quantification was performed manualy using ImageJ. The signal intencity was measured allong the polar and opposite to polar domain (or outer lateral for PIN2 and upper laterl for PIN1 domains as closest to opposite, see Figure 1). After photobleaching (b) the reminecsence of the signal at basal/lateral domains was measured and subtracted from the following timepoints 30, 60, 90, 120, 150, 180min.The FRAP recovery data were analyzed after normalization based on the equation *In=((It-Imin1)/(It2))*100*, where *In* is the normalized intensity; *It* the intensity at any time after bleaching; *Imin1*, the minimum postphotobleaching intensity of the bleached cell; and *It2*, the postphotobleaching intensity of the adjacent nonbleached cell at any time. Based on total FRAP experiments (signal recovery rate in percent) we estimated secretion value for GFP-ABCG37 ~ 0.005 min^{-1}. Because, all tested markers, except PIN2-GFP, showed comparably high secretion rates, parameters obtained for GFP-ABCG37 were considered as representative.

FRAP on Multiple Cells

To examine FRAP within multiple cells, a Spinning Disc System with inverted Zeiss observer, C-Apochromat 63x/1.2 water objective was used to perform high laser power photobleaching for 1.5 to 2 mins in selected ROIs, through approximately 22μm Z-stacks in 45 planes. A Leica SP5 inverted multiphonton microscope was used to collect pre-bleach and post-bleach images to monitor fluorescence recovery at different time points in Z-stacks under the same settings. For quantification, images representing the same layer within the root were extracted from Z-stacks, and the Basal/Lateral index was quantified manually with the line function of ImageJ or by Fiji-Macros software. Note,the background was measured and subtracted from each image individually in figure S13E, and the background signal was not substracted in figure S13D, and the subtraction of different levels of background from different microscopes might lead to the varibility of absolute values of polarity index as compared to the whole cell FRAP analysis.

Protoplasting and Partial Degradation of the Cell Wall

A fresh protoplasting solution was prepared as follows: 1.25% cellulase (Yakult Farmaceutical Ltd.), 0.3% macerozyme (Yakult Farmaceutical Ltd.), 0.4 M D-mannitol (Sigma-Aldrich), 20 mM *2-(N-*morpholino*)*ethanesulfonic acid monohydrate (Duchefa Biochimie), and 20 mM KCl (Merck). We adjusted the pH to 5.7 with 1 M Tris-HCl (pH 7.5) (Invitrogen). The solution was first warmed up at 55°C for 10 min, then cooled down to room temperature. Before use, $CaCl_2$ (Sigma-Aldrich) was added to a final concentration of 10mM. For partial degradation of the cell wall, the cellulose was omitted from the mixture.

Computer Model Description

We adapted a computer model from the framework for the maintenance of PIN polar domains in plant cells recently proposed (Kleine-Vehn et al., 2011; Wabnik et al., 2011) extended it to account for other polar cargos in plants that were analyzed here (PIN1, PIN2, ABCG37, ABCG36, and BOR1). In model simulations, we used root stele cells as a reference. Virtual cell contains apical, basal lateral sides. The PM was abstracted as a sequence of discrete fragments, each of 1×1 micron in size as previously described (Kleine-Vehn et al., 2011; Wabnik et al., 2011). The lateral cell sides were considered twice as long as those of the apical or basal cell sides and resemble the geometry of root stele cells. For simplicity, the intracellular compartments were modeled as one single endosomal compartment that represents an intracellular pool of polar cargo proteins. (based on total FRAP experiments) a constant polar cargo production (0.005 s^{-1}) and estimated degradation rates (0.0001 s^{-1}). The redistribution of polar cargos between PM fragments and endosomal compartments was determined by the basal PIN turnover rates (k_{exo} and k_{endo}). This basal exocytosis and endocytosis rates were set as constant for all cell sides as described in Kleine-Vehn et al. (2011) and Wabnik et al. (2011). Polar cargos displayed a lateral diffusion within the PM (parameter D_m). Definition of parameters is the same as in our previous study Kleine-Vehn et al. (2011) and Wabnik et al., (2011).

We modeled this process by assuming an increased polar cargo delivery rate (k_{SPEX}) to the center of the basal polar domain. We considered two cases: (i) non-polar, *de novo* secretion of polar cargos (k_{exo}), presumptive protein modification signal at the PM that was absent from previous study Kleine-Vehn et al., (2011) and Wabnik et al., (2011) and include the cargo phosphorylation rate (m_{mod}), and subsequent polar recycling of these proteins to the polar domains (integrated in k_{endo}) and (ii) polar secretion of PIN proteins (integrated in k_{SPEX}). These post-translational modifications of cargo proteins were integrated in our previous model Kleine-Vehn et al., (2011) Wabnik et al., (2011) where $CARGO_i^{mod}$ is the pool of phosphorylated cargos in the *i*-th membrane

fragment and $CARGO_i^{nmod}$ describes the non-phosphorylated cargo pool (*de novo* synthesized proteins).

In "polar secretion model" the preferential polar delivery of PIN proteins occurred in a central region within the basal side of the cell with the rate of k_{SPEX}. . The polar PM proteins displayed specific distributions within the PM, namely recruitment into immobile PM microdomains (clusters). The immobile and mobile fractions of polar cargos in the membrane fragments were described by the parameters f_1 and f_2, respectively. For the full description of these model parameters and corresponding equations, we refer to our previous study Kleine-Vehn et al., (2011) and Wabnik et al., (2011).

Model Parameters

In the model simulations, the essential parameter values such as diffusion and secretion rates were estimated from experimental data, D_m = 0.145 $\mu m^2 * s^{-1}$ ± 0.0597 and k_{SPEX} = 0.005 min^{-1}, respectively. In control experiments, the parameters k_{SPEX} and D_m varied up to three orders of magnitude from default values to predict the profiles of the PIN protein distributions in the PM (Figures 8A and 8B). The cargo turnover rates at particular polar domain were predicted for ~30 min half-time of cargo turnover and were $k_{exo} = k_{endo} = \ln(2)/T_{1/2} \sim 0.0005\ s^{-1}$. Non-mobile and mobile protein fractions were $f_1 \sim 0.20$ and $f_2 \sim 1- f_1$ (reflecting relatively high mobility of all tested markers except PIN2, which showed much lower lateral diffusion and stronger clustering) and f_2 varied from 0.1 to 0.9 in simulations presented in Supplementary Figure S14 , respectively. Assumed degradation rate was 0.0001 s^{-1}.
