# Supplementary material for: Cellular mechanisms for cargo delivery and polarity maintenance at different polar domains in plant cells
Source: Cell Discov. 2016 Jul 19;2:16018–. doi: 10.1038/celldisc.2016.18 (PMC4950145; doi:10.1038/celldisc.2016.18)
Supplement: Supplementary Figure S1 [file celldisc201618-s2.pdf]

SFigure 1

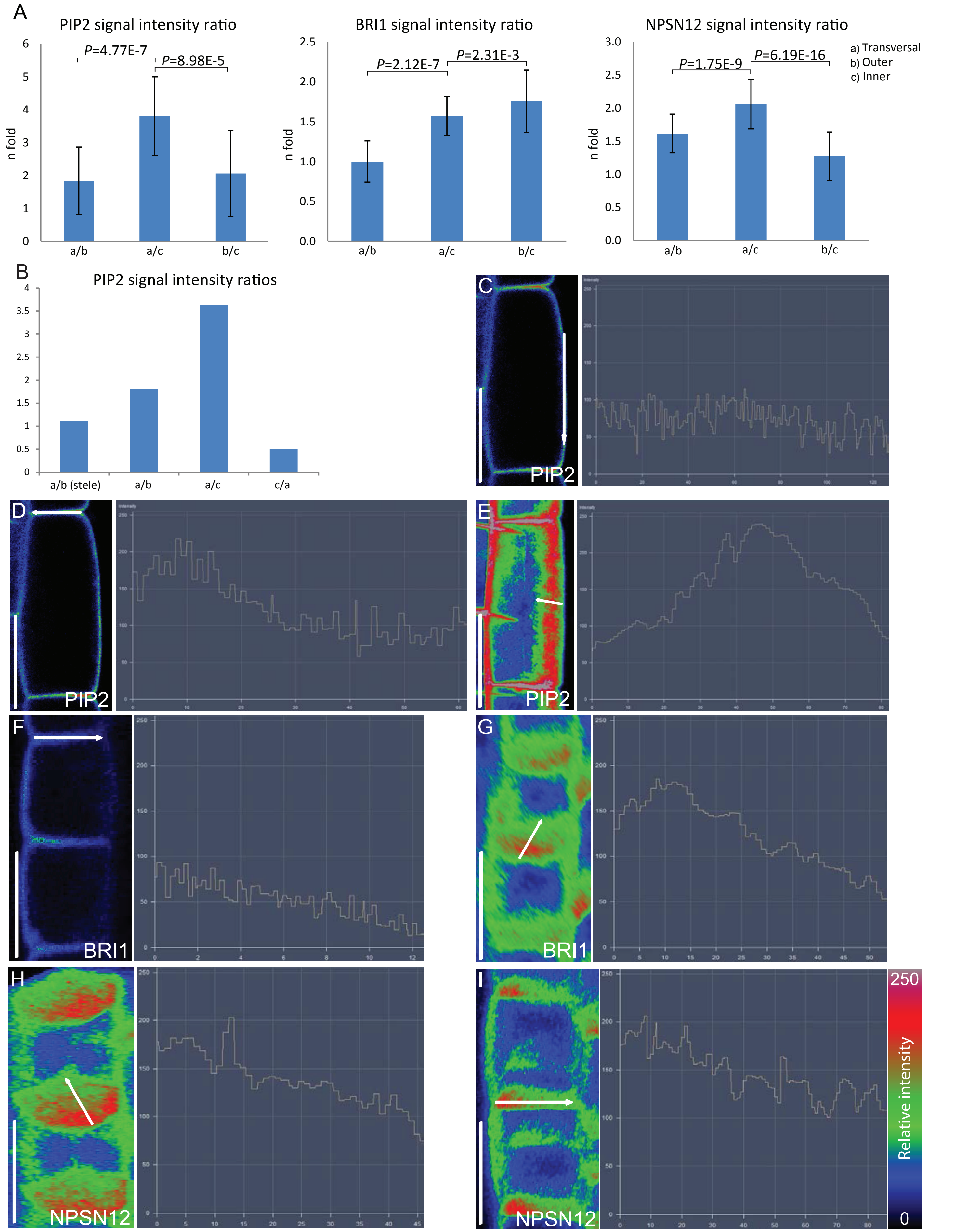

Supplemental Figure 1. Comparative Analysis of Signal Distribution of Various Apolar Markers.
(A) Quantification of the maximal polarity index for steady-state PIP2-GFP, BRI1-GFP and YFP-WAVE131 between transversal (a), outer (b) and inner (c) domains.
(B) Quantification of the maximal polarity index for the steady-state PIP2-GFP. All ratios were calculated using values measured in epidermal cells except first ratio a/b (stele) that is based on values measured in the stele cells. (C-H) Median optical sections (C, D and F) and 3D xyz projections (0.4 µm step) (E and G-I) of epidermal cells expressing PIP2-GFP (C-E), BRI1-GFP (F and G), and YFP-NPNS12 (H and I). All markers displayed somewhat asymmetric protein localization, with a stronger signal intensity at transversal domain (merged apical and basal), dispersing from one end of the domain (adjacent to outer) to other (adjacent to inner). The right-hand panels represent the signal intensity distribution analysis along the white arrows. Fluorescence intensity from 0 (black) to 250 (bright/white) is represented by the color code. Scale bar = 20µm.
