# Supplementary material for: Cellular mechanisms for cargo delivery and polarity maintenance at different polar domains in plant cells
Source: Cell Discov. 2016 Jul 19;2:16018–. doi: 10.1038/celldisc.2016.18 (PMC4950145; doi:10.1038/celldisc.2016.18)
Supplement: Supplementary Figure S2 [file celldisc201618-s3.pdf]

SFigure 2

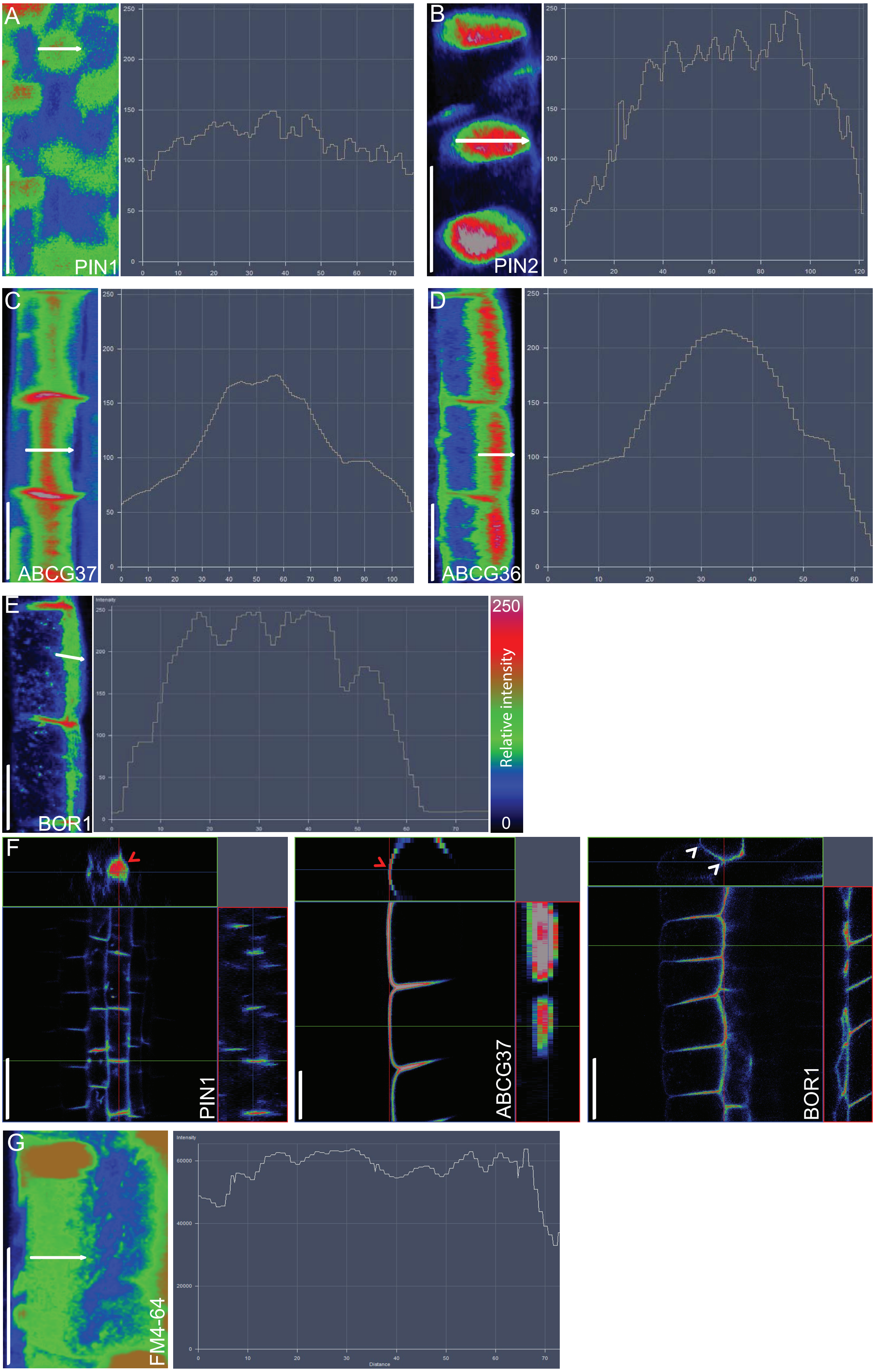

Supplementary Figure 2. Polar Markers Distribution at Cellular Level in 3D.

(A-E) Analysis of projected 3D reconstructions (0.4 µm steps) of stele cells expressing PIN1-GFP (basal) (A), epidermal cells expressing PIN2-GFP (apical) (B), ABCG36-GFP (outer) (C), GFP-ABCG37 (outer) (D), BOR1-GFP (inner) (E) displaying clear asymmetry in protein localization in the respective polar domains. In the left panels are the respective projections and in the right panels, the intensity profiles are shown across the arrows of the respective projections. In general a peak in signal intensity can be observed. (F) Analysis of projected 3D reconstructions (0.4 µm steps) of stele and epidermal cells expressing PIN1-GFP (basal), GFP-ABCG37 (outer), BOR1-GFP (inner). Mid (blue frame) rectangles represent front view; top (green frame) rectangles represent a vertical section (view from the top); right (red frame) rectangles represent side view (90 degrees rotated in regard to the mid rectangle). Red arrows indicate signal enrichment in the central zone of the PM domain, white arrows asymmetric signal distribution of BOR1-GFP, decreasing along the proximal domain, from the inner towards outer domain. (G) Analysis of projected 3D reconstruction (0.4 µm steps) of epidermal cell 10min treated with 4 µM FM4-64 (left panel). Right panel represents the intensity profile across the arrow drawn horizontally across the outer lateral domain. The pattern is significantly different from patterns obtained for the GFP-tagged proteins GFP-ABCG37 and AGCG36-GFP. Fluorescence intensity from 0 (black) to 250 (bright/white) is represented by the color code. Scale bar = 20µm.
