# Supplementary material for: Cellular mechanisms for cargo delivery and polarity maintenance at different polar domains in plant cells
Source: Cell Discov. 2016 Jul 19;2:16018–. doi: 10.1038/celldisc.2016.18 (PMC4950145; doi:10.1038/celldisc.2016.18)
Supplement: Supplementary Figure S3 [file celldisc201618-s4.pdf]

SFigure 3

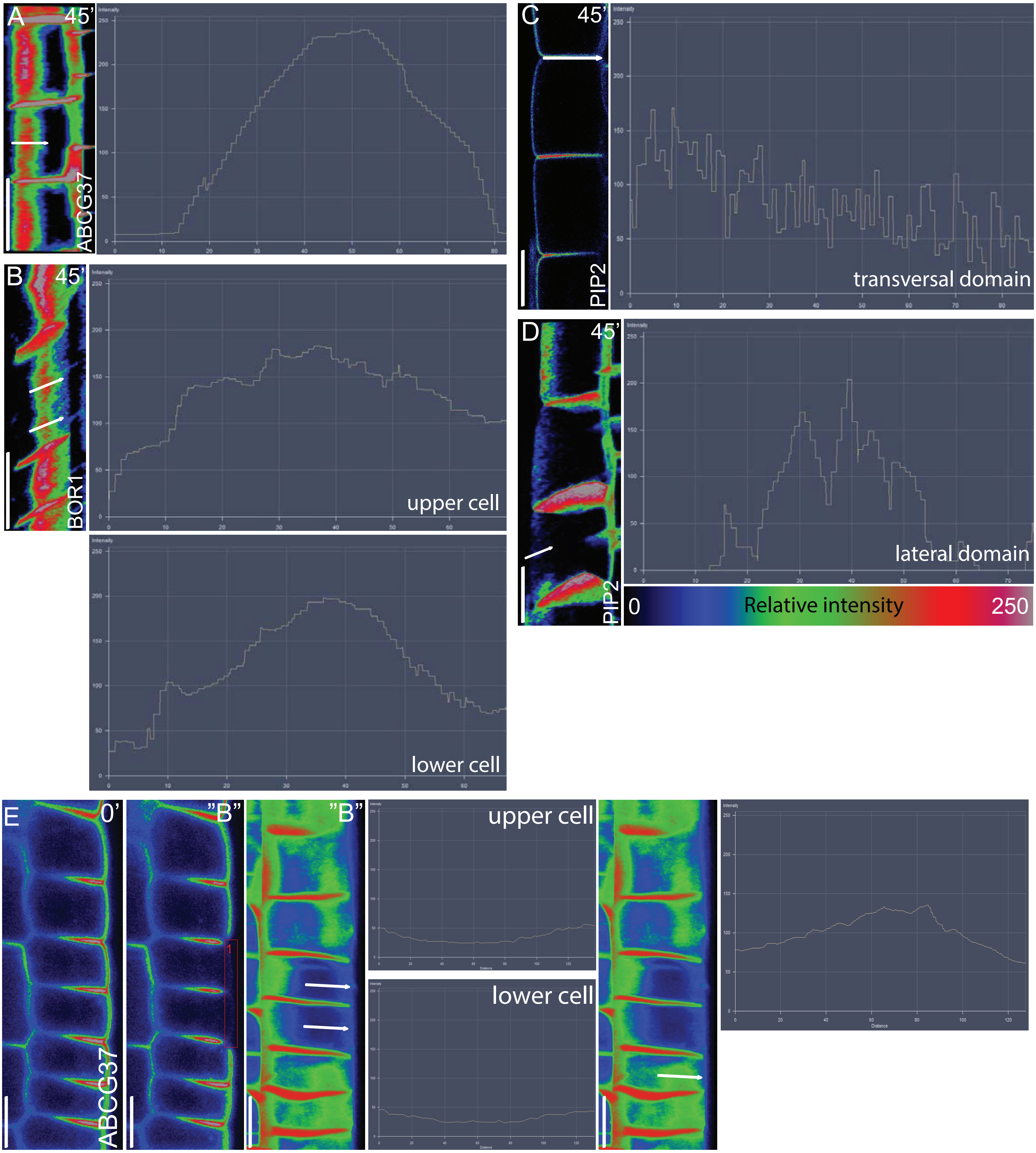

Supplementary Figure 3. Super-Polar Recycling of Markers Defining Lateral Polar Domain.
(A-D) Fluorescence profile analysis (along the indicated white arrows) on 3D projections (0.4 µm step size) of cell 45 min after photobleaching (see corresponding Figure 2) for GFP-ABCG37 (A), BOR1-GFP (B), and PIP2-GFP (C and D).
(E) Control of the bleaching efficiency for most intensive fluorescent marker ABCG37-GFP. Signal intencity before (0') and after bleaching ("B"). Fluorescence intensity from 0 (black) to 250 (bright/white) is represented by the color code. Scale bar = 20µm.
