# Supplementary material for: Cellular mechanisms for cargo delivery and polarity maintenance at different polar domains in plant cells
Source: Cell Discov. 2016 Jul 19;2:16018–. doi: 10.1038/celldisc.2016.18 (PMC4950145; doi:10.1038/celldisc.2016.18)
Supplement: Supplementary Figure S4 [file celldisc201618-s5.pdf]

SFigure 4

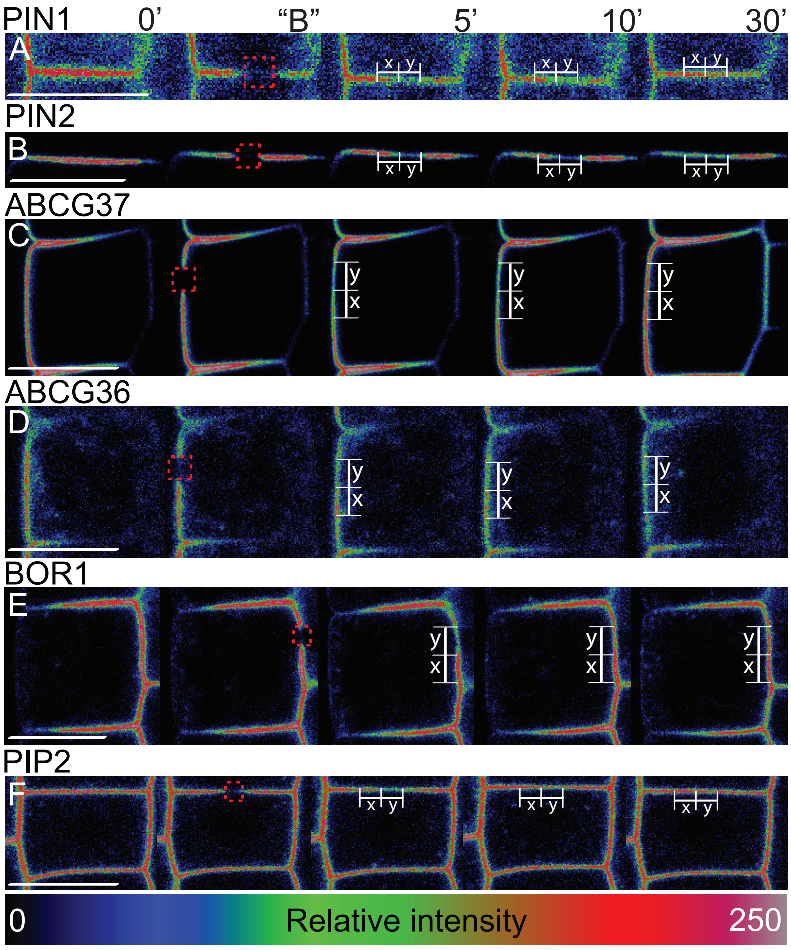

Supplementary Figure 4. Additive Effect of Lateral Diffusion, Secretion and Recycling on Signal Recovery of Photobleached 2-µm PM Region. (A-F) FRAP of a 2-µm PM region (dashed red rectangle) done on the respective polar domains of PIN1-GFP (basal) (A), PIN2-GFP (apical) (B), GFP-ABCG37 (outer) (C), ABCG36-GFP (outer) (D), BOR1-GFP (inner) (E), and PIP2-GFP (apical) (F), with indication of the bleached area (red rectangles), and the regions 'x' and 'y' which were used to estimate the relative signal recovery rates in Figure3. Prebleaching (0'), postbleaching ("B"), and three recovery time points (5, 10, and 30 min) were recorded. Fluorescence intensity from 0 (black) to 250 (bright/white) is represented by the color code. n=4-5 FRAP experiments on different roots. Scale bar 10 µm.
