# Supplementary material for: Cellular mechanisms for cargo delivery and polarity maintenance at different polar domains in plant cells
Source: Cell Discov. 2016 Jul 19;2:16018–. doi: 10.1038/celldisc.2016.18 (PMC4950145; doi:10.1038/celldisc.2016.18)
Supplement: Supplementary Figure S5 [file celldisc201618-s6.pdf]

SFigure 5

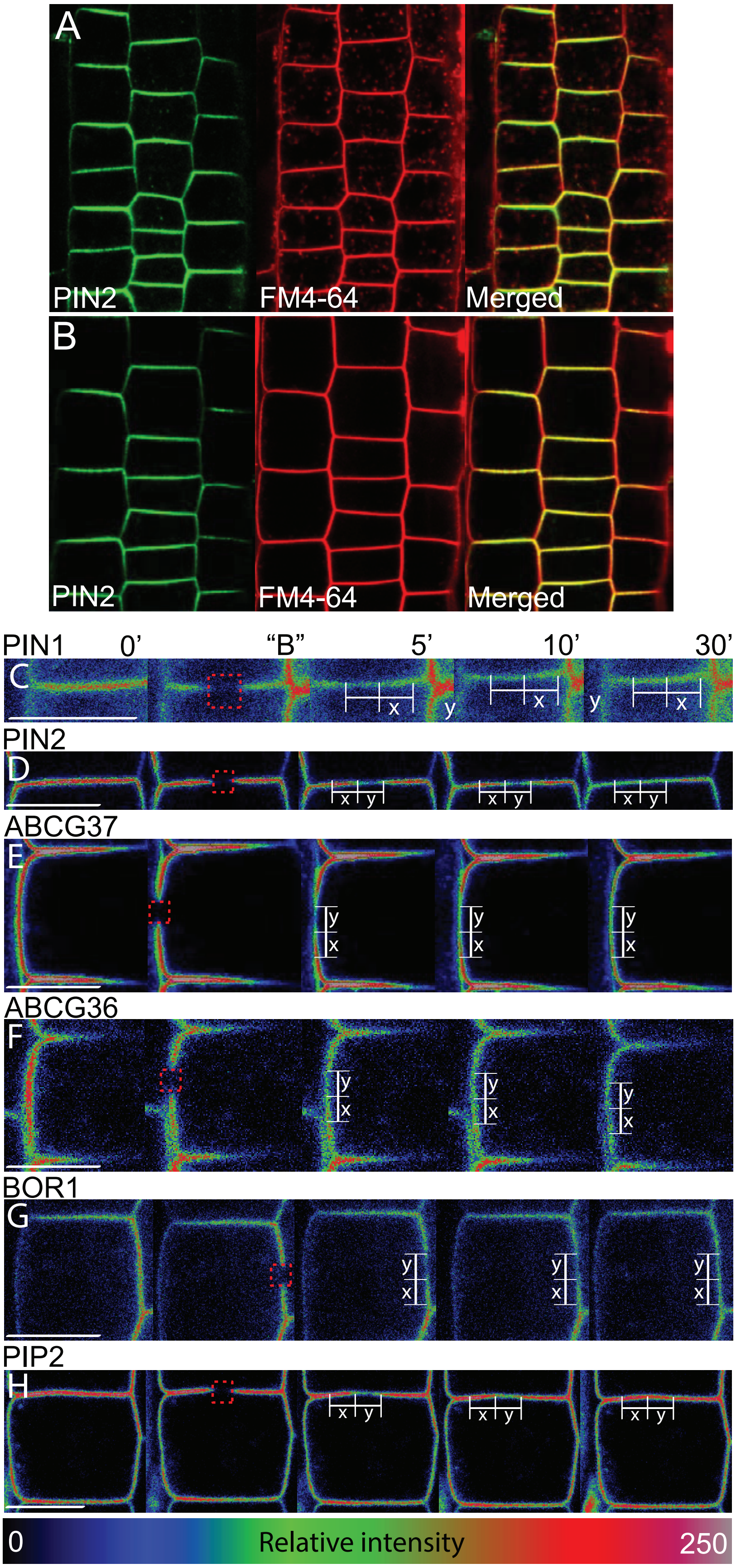

Supplementary Figure 5. Lateral Diffusion Effect on the Signal Recovery of the Photobleached 2-µm PM Region.
(A and B) Visualisation of endocytotic trafficking via FM4-64 (4µM) on PIN2-GFP roots, under control conditions (A) and after pretreatment with energy, callose, and biosynthesis inhibitors (-e) (0.02% sodium azide, 50 mM 2-deoxy-D-glucose, and 50 µm CHX) (B). Note the lack of intracellular FM4-64 staining in (B).
(C-H) local FRAP analysis under these conditions for PIN1-GFP (C), PIN2-GFP (D), GFP-ABCG37 (E), ABCG36-GFP (F), BOR1-GFP (G), and PIP2-GFP (H), with indication of the regions ‘x’ and ‘y’ which were used to estimate the relative signal recovery rates in Figure 3. Prebleaching (0'), postbleaching ("B"), and three recovery time points (5, 10, and 30 min) were recorded. Fluorescence intensity from 0 (black) to 250 (bright/white) is represented by the color code. n=4-5 FRAP experiments on different roots. Scale bar 10 µm.
