# Supplementary material for: Cellular mechanisms for cargo delivery and polarity maintenance at different polar domains in plant cells
Source: Cell Discov. 2016 Jul 19;2:16018–. doi: 10.1038/celldisc.2016.18 (PMC4950145; doi:10.1038/celldisc.2016.18)
Supplement: Supplementary Figure S6 [file celldisc201618-s7.pdf]

SFigure 6

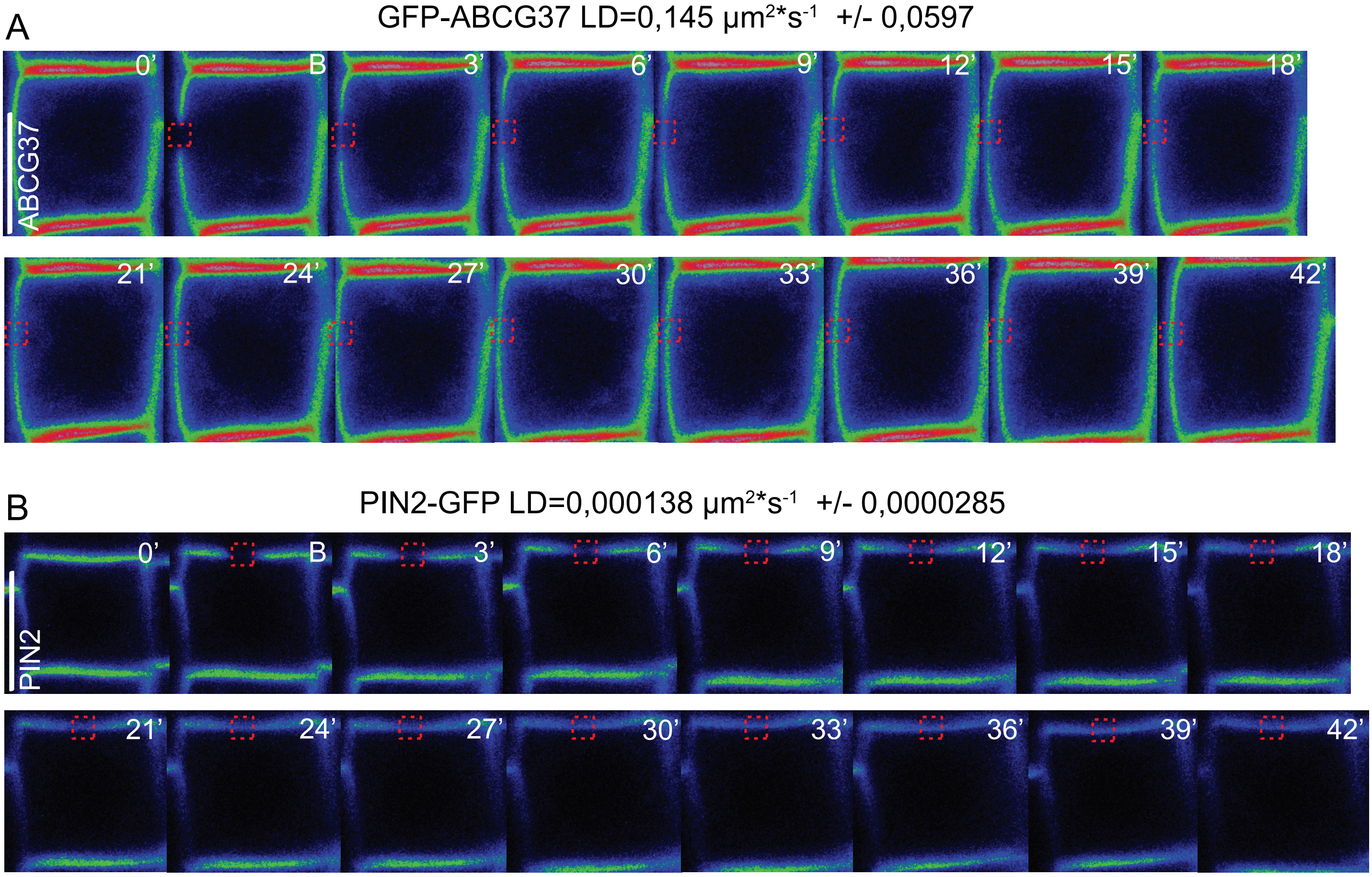


**Supplementary Figure 6.** Protein Lateral Diffusion Efficiency of GFP-ABCG37 and PIN2-GFP Differs More Than 1000-Fold.
(A and B) Photobleaching and recovery of the 2-µm PM region were performed for GFP-ABCG37 (A) and PIN2-GFP (B). Every 3 min until 42 min recovery progression was recorded. Basing on FRAP results we generated a parameter of protein lateral diffusion for GFP-ABCG37 (LD = 0.145 µm2*s-1 +/-0.0597) and PIN2-GFP (D = 0.000138 +/- 0.0000285) (for more details see Chen et al, 2006). Parameters were generated basing on three independent repetitions. Fluorescence intensity from 0 (black) to 250 (bright/white) is represented by the color code. Scale bar 10 µm.
