# Supplementary material for: Cellular mechanisms for cargo delivery and polarity maintenance at different polar domains in plant cells
Source: Cell Discov. 2016 Jul 19;2:16018–. doi: 10.1038/celldisc.2016.18 (PMC4950145; doi:10.1038/celldisc.2016.18)
Supplement: Supplementary Figure S7 [file celldisc201618-s8.pdf]

SFigure 7

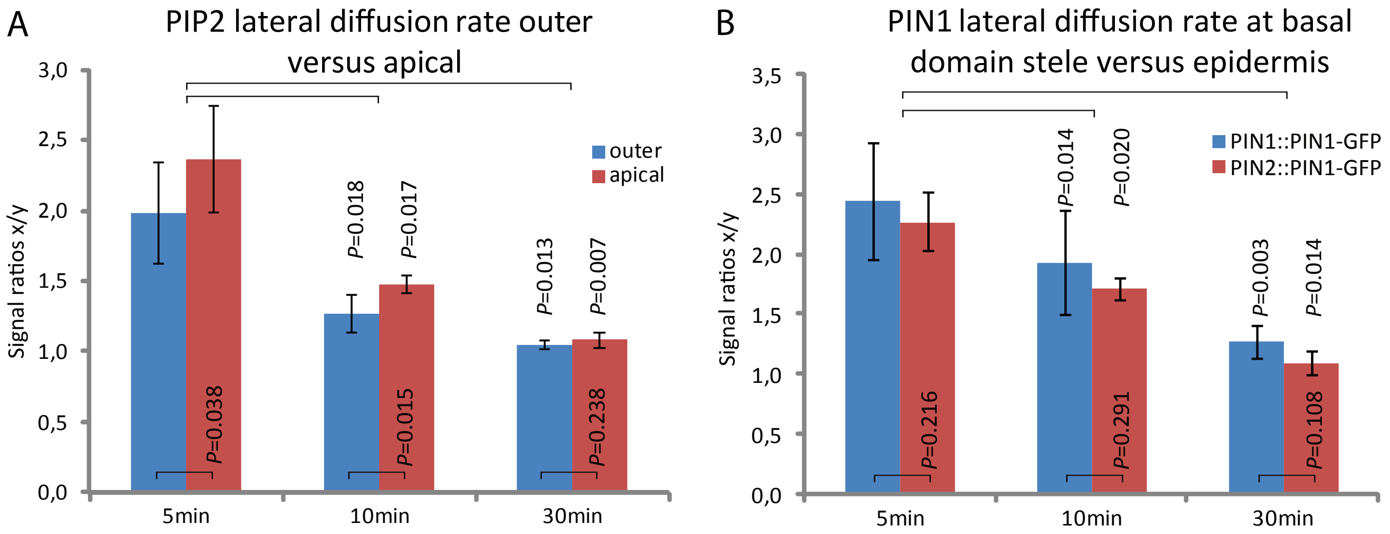

Supplementary Figure 7. Impact of Different Polar Cargo Localizations and Cell Types on the Lateral Diffusion Rate.
(A-B) Comparison of relative signal recovery rates of PIP2 at the outer and apical polar domains (A). Comparison of relative signal recovery rates between PIN1-GFP in the stele (PIN1::PIN1-GFP) and in the epidermis (PIN2::PIN1-GFP) (B). The signal values of prebleach and postbleach fluorescence intensities were normalized and averaged. Error bars represent standard error of the mean (s.e.m), P-value calculated according to Student's t-test. n=4-5 FRAP experiments on different roots.
