# Supplementary material for: Cellular mechanisms for cargo delivery and polarity maintenance at different polar domains in plant cells
Source: Cell Discov. 2016 Jul 19;2:16018–. doi: 10.1038/celldisc.2016.18 (PMC4950145; doi:10.1038/celldisc.2016.18)
Supplement: Supplementary Figure S8 [file celldisc201618-s9.pdf]

SFigure 8

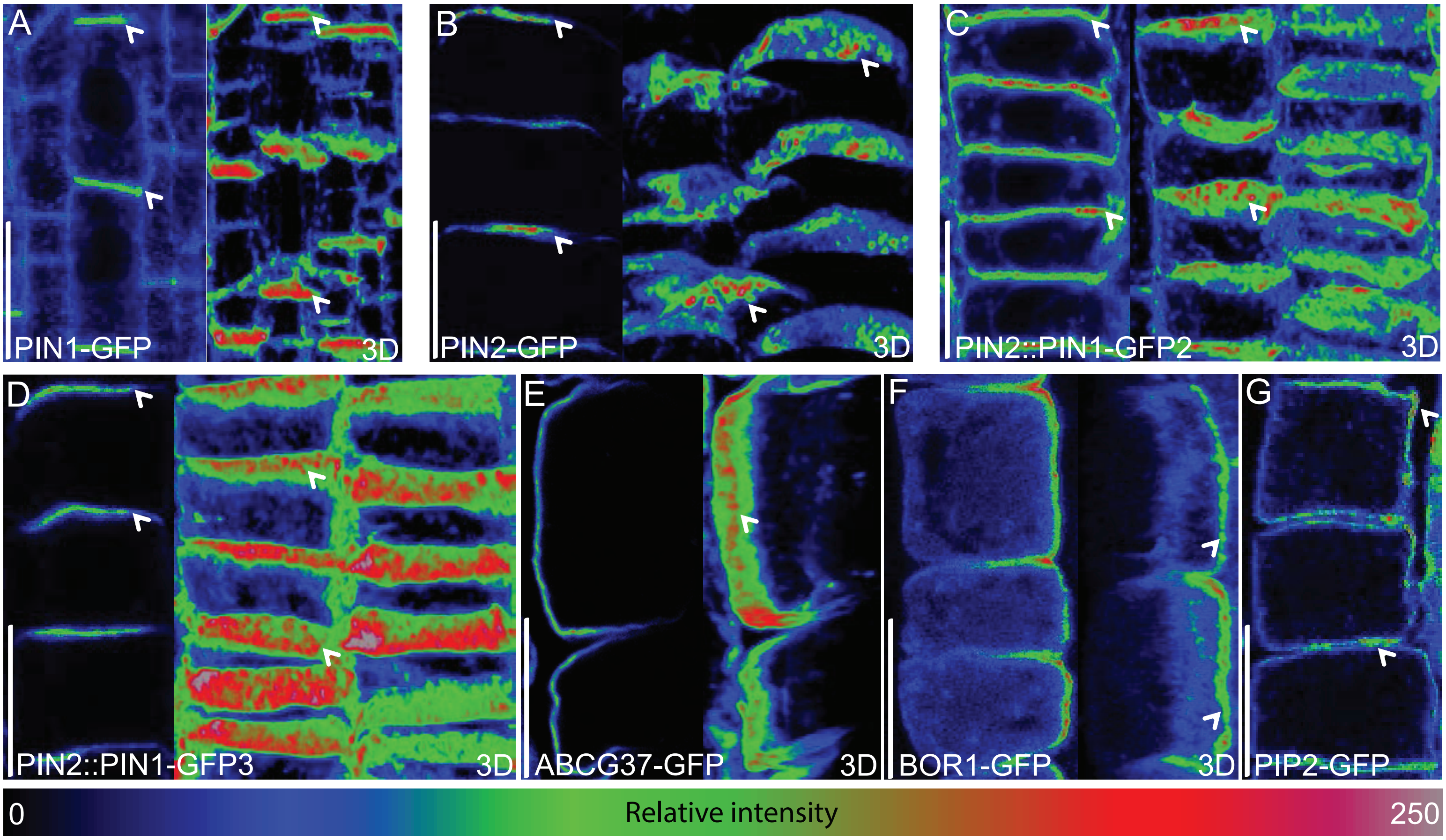

Supplementary Figure 8. Visualization of Clusters for Various Polar Markers after Fixation.
(A-G) Confocal microscopy of PFA (paraformaldehyde) fixed samples reveals protein clustering (indicated by white arrows) of PIN1::PIN1-GFP (basal; stele) (A), PIN2::PIN2-GFP (apical; epidermis) (B), PIN2::PIN1-GFP2 (basal; epidermis) (C), PIN2::PIN1-GFP3 (apical; epidermis) (D), GFP-ABCG37 (outer-lateral; epidermis) (E), BOR1-GFP (inner-latter; epidermis) (F), and PIP2-GFP (epidermis). PIN1-GFP expressed in epidermis shows more pronounced fragmented protein crowding accumulation in comparison to PIN1-GFP expressed in stele. GFP-ABCG37 and BOR1-GFP show weaker clustering degree comparing to PIN proteins expressed in epidermis (see also Supplementary Figure 11). 3D depicts xyz projections (step size 0.4 µm). Fluorescence intensity from 0 (black) to 250 (bright/white) is represented by the color code. Scale bar 20 µm.
