# Supplementary material for: Cellular mechanisms for cargo delivery and polarity maintenance at different polar domains in plant cells
Source: Cell Discov. 2016 Jul 19;2:16018–. doi: 10.1038/celldisc.2016.18 (PMC4950145; doi:10.1038/celldisc.2016.18)
Supplement: Supplementary Figure S9 [file celldisc201618-s10.pdf]

SFigure 9

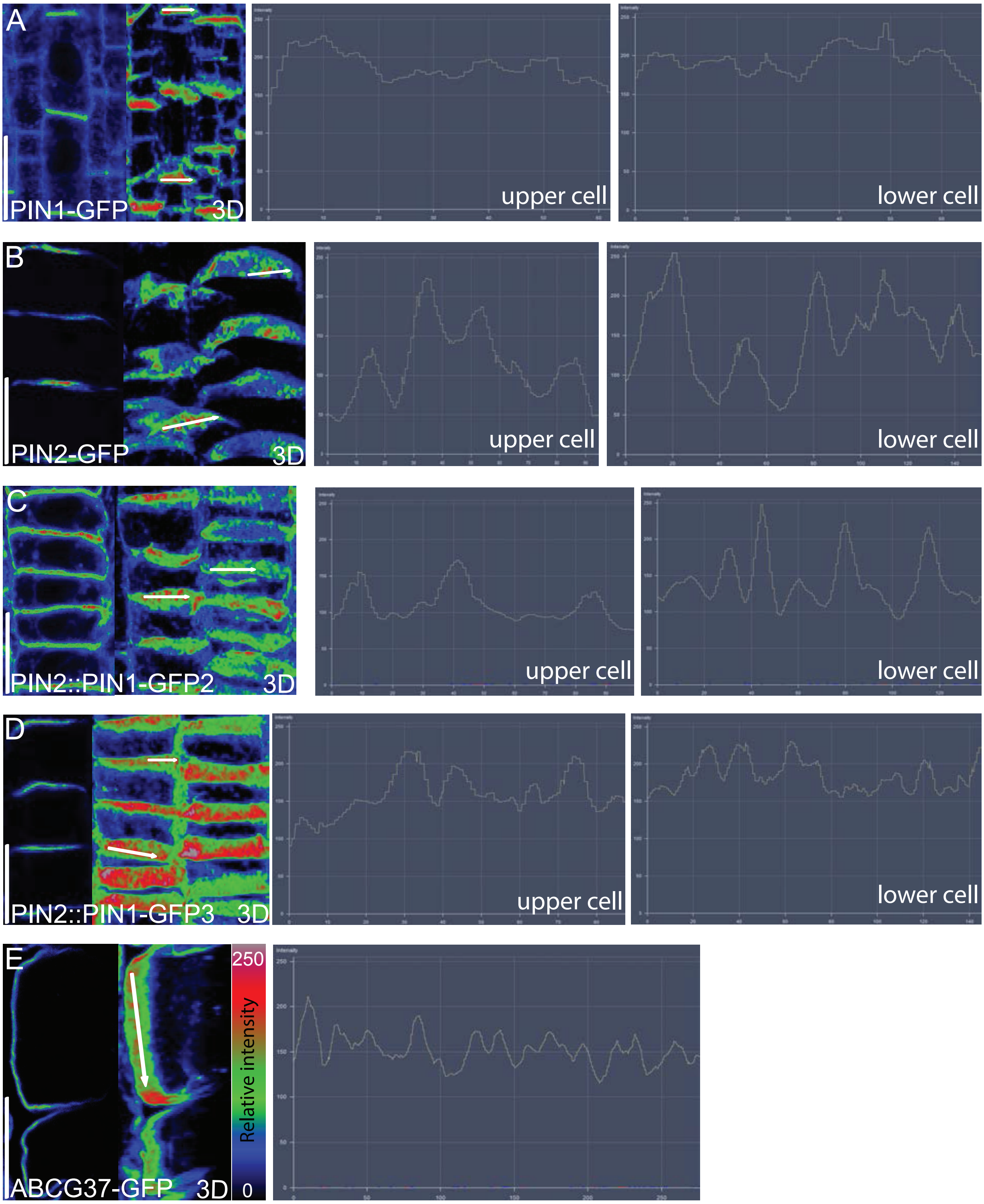

Supplementary Figure 9. Protein Clustering Degree Differentiates Between Polar Markers.
(A-E) Fluorescence profile analysis along the indicated white arrows for PIN1-GFP in the stele (A), or epidermis (C and D), for PIN2 in the epidermis (B) and GFP-ABCG37 in the epidermis (E) 3D depicts xyz projections (step size 0.4 µm). Fluorescence intensity from 0 (black) to 250 (bright/white) is represented by the color code. Scale bar 20 µm.
