# Supplementary material for: Cellular mechanisms for cargo delivery and polarity maintenance at different polar domains in plant cells
Source: Cell Discov. 2016 Jul 19;2:16018–. doi: 10.1038/celldisc.2016.18 (PMC4950145; doi:10.1038/celldisc.2016.18)
Supplement: Supplementary Figure S10 [file celldisc201618-s11.pdf]

SFigure 10

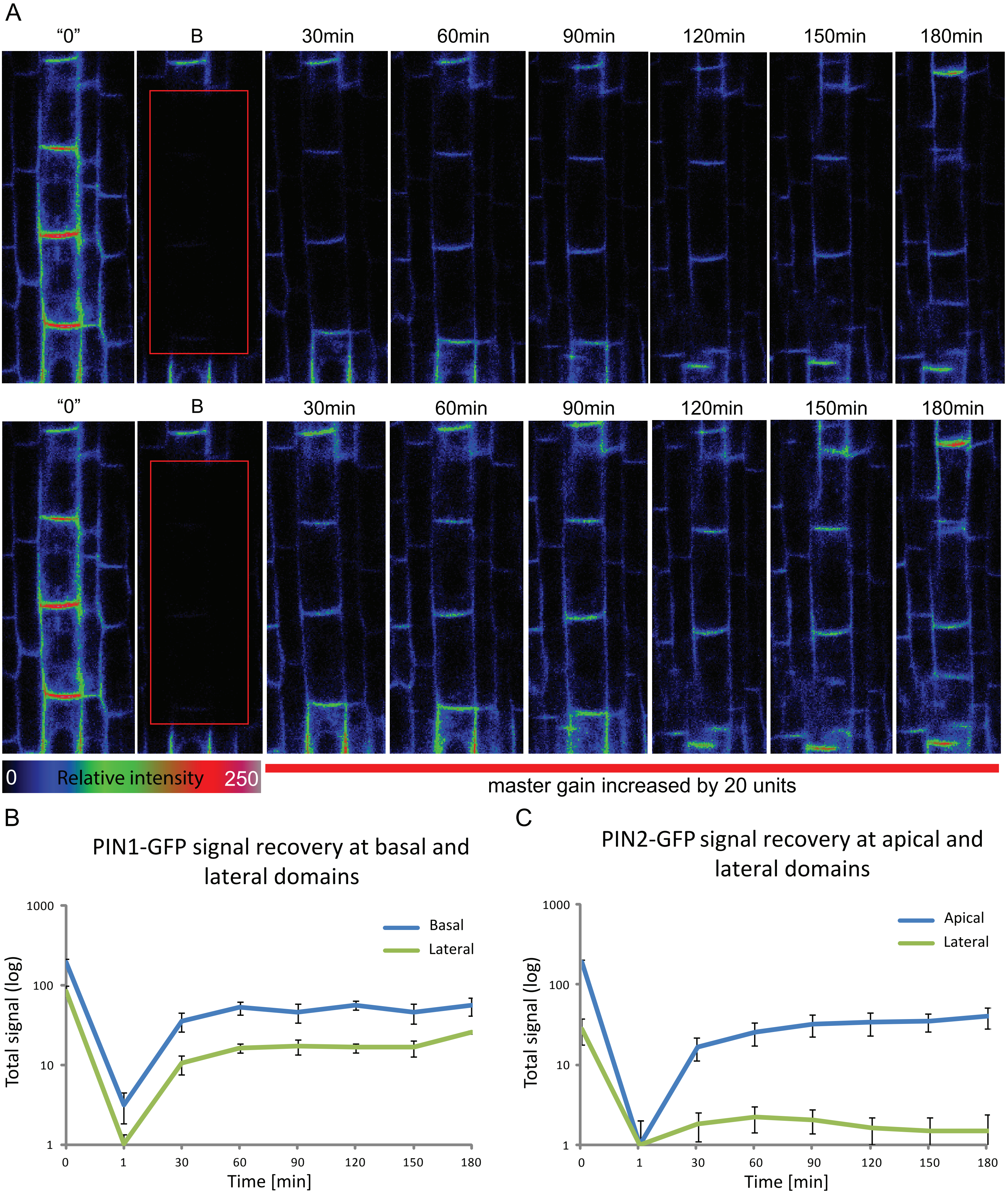

Supplementary Figure 10. Whole Cell FRAP Analysis for PIN1-GFP and PIN2-GFP.
(A) Full projection of PIN1-GFP recovery after photobleaching a complete cell shows continuous protein delivery to basal domain (A). Upper panel shows the FRAP without adjusting the master gain. Lower panel shows the same FRAP experiment with an increase master gain of 20 units after bleaching. Because during FRAP experiment, both bleached and non-bleached cells show depleted signal intensity, increase in gain allows better measurements and quantification of signal ratio between polar and non-polar domains. Red rectangle indicates bleached area. Prebleaching (0'), postbleaching ("B"), and subsequent images illustrating recovery time points every 30 min until 180 min were recorded.
(B and C) Quantitative analysis of PIN1-GFP (B) and PIN2-GFP (C) signal recovery in logarithmic scale display different pattern of protein secretion and recycling. The signal values of prebleach and postbleach fluorescence intensities were normalized and are standard error of mean ± (s.e.m). n=8 FRAP experiments on different roots. Fluorescence intensity from 0 (black) to 250 (bright/white) is represented by the color code. Scale bar 20 µm.
