# Supplementary material for: Cellular mechanisms for cargo delivery and polarity maintenance at different polar domains in plant cells
Source: Cell Discov. 2016 Jul 19;2:16018–. doi: 10.1038/celldisc.2016.18 (PMC4950145; doi:10.1038/celldisc.2016.18)
Supplement: Supplementary Figure S11 [file celldisc201618-s12.pdf]

SFigure 11

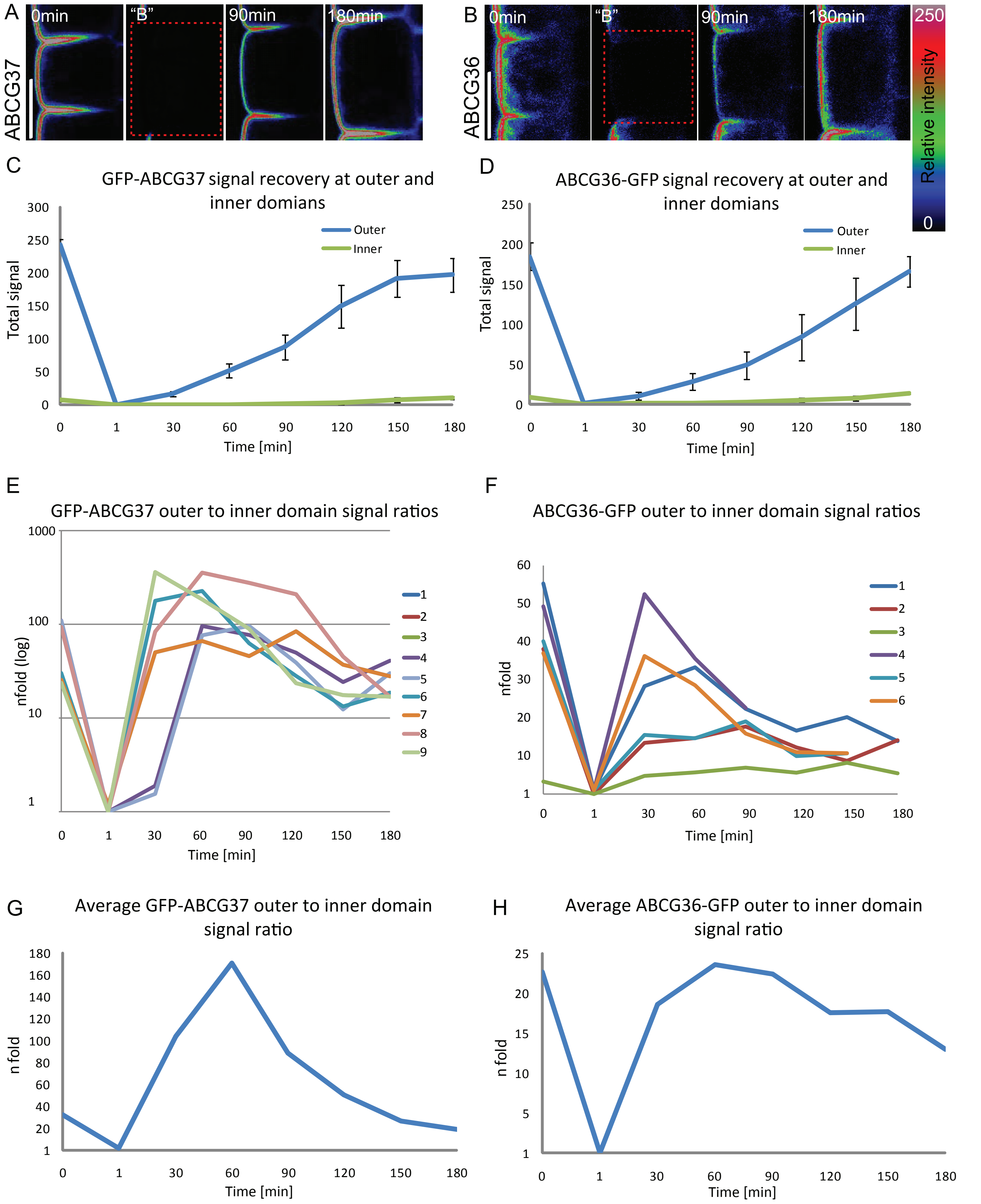

Supplementary Figure 11. Whole Cell FRAP Analysis for GFP-ABCG37 and ABCG36-GFP.
(A and B) Fluorescence recovery after whole cell photobleaching for outer lateral markers GFP-ABCG37 (A) and ABCG36-GFP (B). Red rectangle indicates bleached region. Fluorescence intensity from 0 (black) to 250 (bright/white) is represented by the color code. Scale bar 10 µm.
(C and D) Evolution of signal intensity GFP-ABCG37 (C) and ABCG36-GFP (D) at outer and inner lateral domains.
(E-H) The signal intensity (outer versus inner) ratios for GFP-ABCG37 (E and G) and ABCG36-GFP (F and H) in extended time frame. In the graphs E and F each colored profile (numbered 1-9) represents the ratio of signal intensities calculated from individual FRAPs experiments. The signal values of the prebleach and postbleach fluorescence intensities data were normalized and error bars are standard error of mean ± (s.e.m). n=6-9 FRAP experiments on different roots.
