# Supplementary material for: Cellular mechanisms for cargo delivery and polarity maintenance at different polar domains in plant cells
Source: Cell Discov. 2016 Jul 19;2:16018–. doi: 10.1038/celldisc.2016.18 (PMC4950145; doi:10.1038/celldisc.2016.18)
Supplement: Supplementary Figure S12 [file celldisc201618-s13.pdf]

SFigure 12

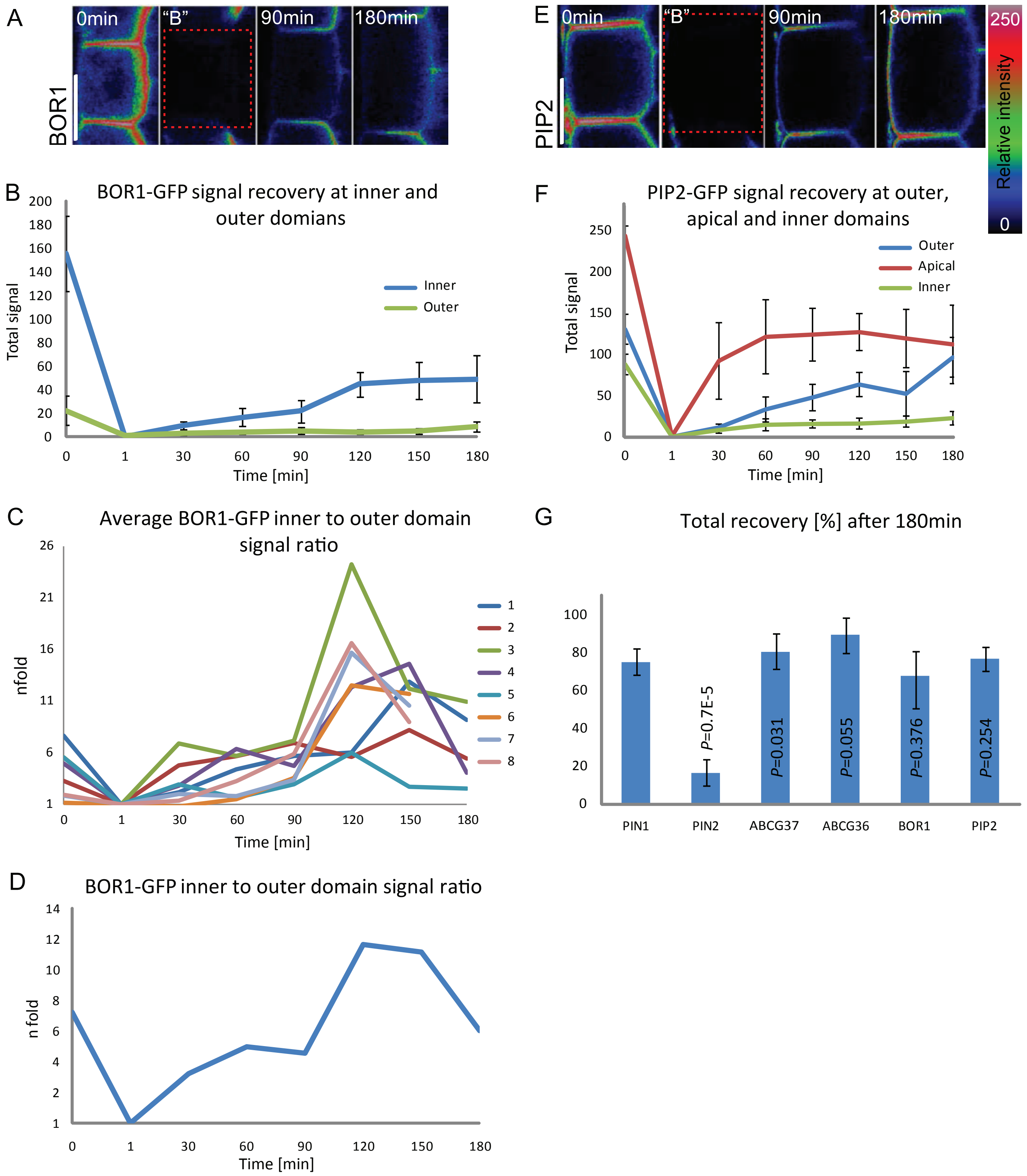

Supplementary Figure 12. Whole Cell FRAP Analysis for BOR1-GFP and PIP2-GFP.
(A-D) Fluorescence recovery after whole cell photobleaching for BOR1-GFP (A). Evolution of the signal intensity for BOR1-GFP at the inner and outer domains (B). (C and D) The signal intensity (inner versus outer) ratios for BOR1-GFP over time (C and D). In the graph C each colored profile (numbered 1-8) represents the ratio of signal intensities calculated from individual FRAP experiments. The signal values of prebleach and postbleach fluorescence intensities were normalized and are standard error of mean averaged (s.e.m). n=4-8 FRAP experiments on different roots.
(E and F) Fluorescence recovery after whole cell photobleaching for non-polar reference marker PIP2-GFP (E). Fluorescence intensity from 0 (black) to 250 (bright/white) is represented by the color code. Scale bar 10 µm. (F) PIP2-GFP recovery profiles for transversal, outer, and inner lateral domains over 180min. The signal values of prebleach and postbleach fluorescence intensities were normalized and error bars are standard error of mean ± (s.e.m). n=4-8 FRAP experiments on different roots.
(G) Total signal recovery of photobleached GFP-fused markers after 180 min, in percent (data were normalized, for the details see Material and Methods). Error bars represent standard error of mean (s.e.m), P-value calculated according to Student's t-test.
