# Supplementary material for: Cellular mechanisms for cargo delivery and polarity maintenance at different polar domains in plant cells
Source: Cell Discov. 2016 Jul 19;2:16018–. doi: 10.1038/celldisc.2016.18 (PMC4950145; doi:10.1038/celldisc.2016.18)
Supplement: Supplementary Figure S13 [file celldisc201618-s14.pdf]

SFigure 13

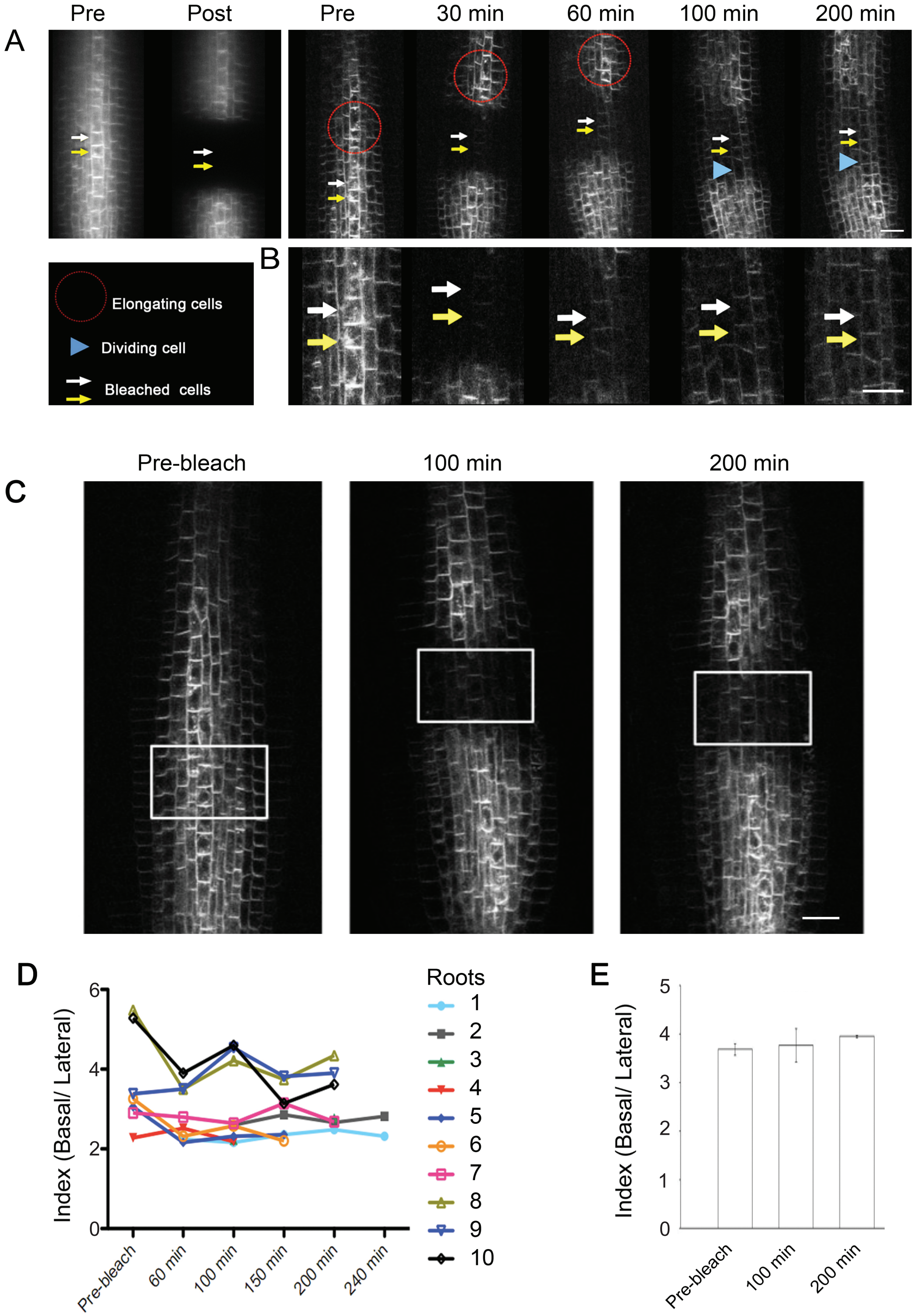

Supplementary Figure 13. Multiple Cells FRAP Analysis of PIN1-GFP by Spinning Disc and 2-photon Microscopy.
(A-C) PIN1-GFP fluorescence recovery images taken under 2-photon microscope after multiple cell photobleaching. Red circles highlight elongating cells, and the blue arrow heads point to dividing cells which indicate continued growth during fluorescence recovery. White and yellow arrows point to identical cells throughout recovery. Scale bars = 20 µm. Pre- and post-bleach images were captured under a spinning disc microscope used to thoroughly photobleach in 3 dimensions (A, left panel); in all other images, fluorescence recovery was monitored under a 2-photon microscope for better florescence detection in the interior of the root and less background fluorescence (A, right panel). Enlargement of (A) tracking fluorescence recovery in selected cells (B). Tracking FRAP in multiple cells after bleaching the central part of the root in 3 dimensions. Images representing the same layer within the root were extracted out of the Z-stacks from different time points (C). White boxes indicate the FRAP area.
(D-E) Quantification of the Basal/Lateral index of PIN1-GFP signal during recovery. Manual analysis of 10 different roots; recovery images of roots 1 to 6 were taken under a spinning disc microscope, while images of roots 7 to10 were taken under a 2-photon microscope (D). The Basal/Lateral index was quantified from Z-stack images representing the same layer within the root at different time points Automated analysis of mean intensity values within the basal or lateral membranes of all cells in FRAP regions. FRAP regions within each image contain roughly 20 cells. 4 sets of Z-stack tracking out of 2 different roots were analyzed (E). n ~80 cells.
