# Supplementary material for: Cellular mechanisms for cargo delivery and polarity maintenance at different polar domains in plant cells
Source: Cell Discov. 2016 Jul 19;2:16018–. doi: 10.1038/celldisc.2016.18 (PMC4950145; doi:10.1038/celldisc.2016.18)
Supplement: Supplementary Figure S14 [file celldisc201618-s15.pdf]

SFigure 14

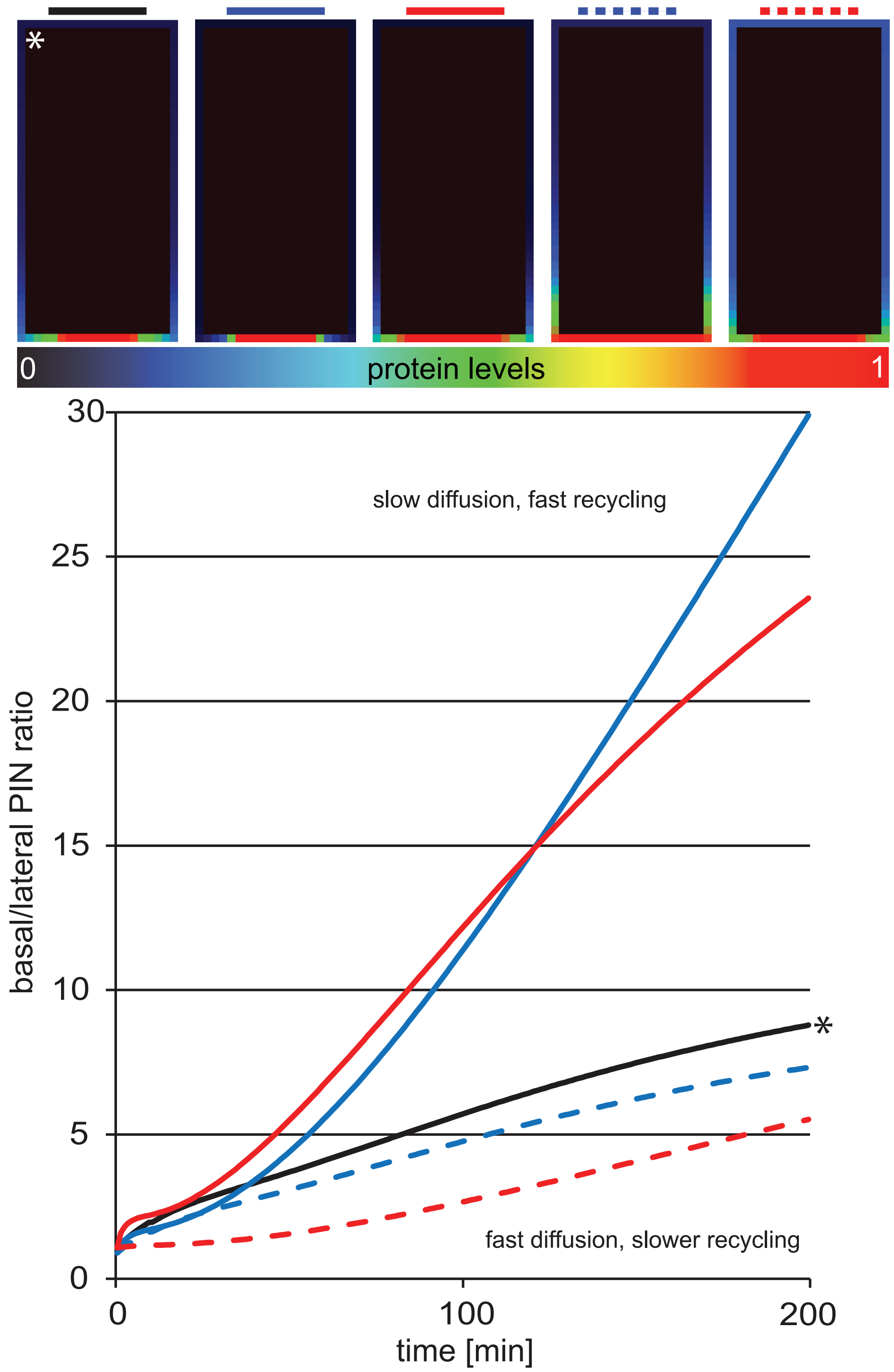

Supplementary Figure 14. Simulations of Both Protein Lateral Diffusion and Recycling on Protein Polarization Dynamics in "Non-Polar Secretion" Model. (A) Computational simulations representing predicted cell polarity that depends on two factors, lateral diffusion and polar recycling, in the non-polar secretion model (upper panel), and chart representing polarity index profiles (signal ratios between polar and non-polar domains) (lower panel). Combination of these two factors is sufficient to generate polar protein distribution and maintain the cell polarity (upper panel). Nevertheless the 'non-polar' secretion model could not predict for any of experimentally observed polarity index profiles of PIN1-GFP, GFP-ABCG37, ABCG36-GFP and BOR1-GFP. Simulations depicted by asterisk (black line) represent reference simulations with experimentally determined parameter values (Dm = 0.145 µm2*s-1 and kendo = 0.00005 min-1).. Blue and solid red lines shows predictions from "non-polar" secretion model with slow lateral diffusion (Dm = 0.00001 µm2*s-1) and fast recycling (kendo = 0.05 min-1), respectively. Accoridingly, blue and red dashed lines shows predicted polarity indexes for fast lateral (Dm = 1 µm2*s-1) difussion and slow recycling (kendo = 0.00005 min-1).Protein levels are represented by color coding scheme, from low (0.0001) to high (1) (log scale).
