# Supplementary material for: Cellular mechanisms for cargo delivery and polarity maintenance at different polar domains in plant cells
Source: Cell Discov. 2016 Jul 19;2:16018–. doi: 10.1038/celldisc.2016.18 (PMC4950145; doi:10.1038/celldisc.2016.18)
Supplement: Supplementary Figure S15 [file celldisc201618-s16.pdf]

SFigure 15

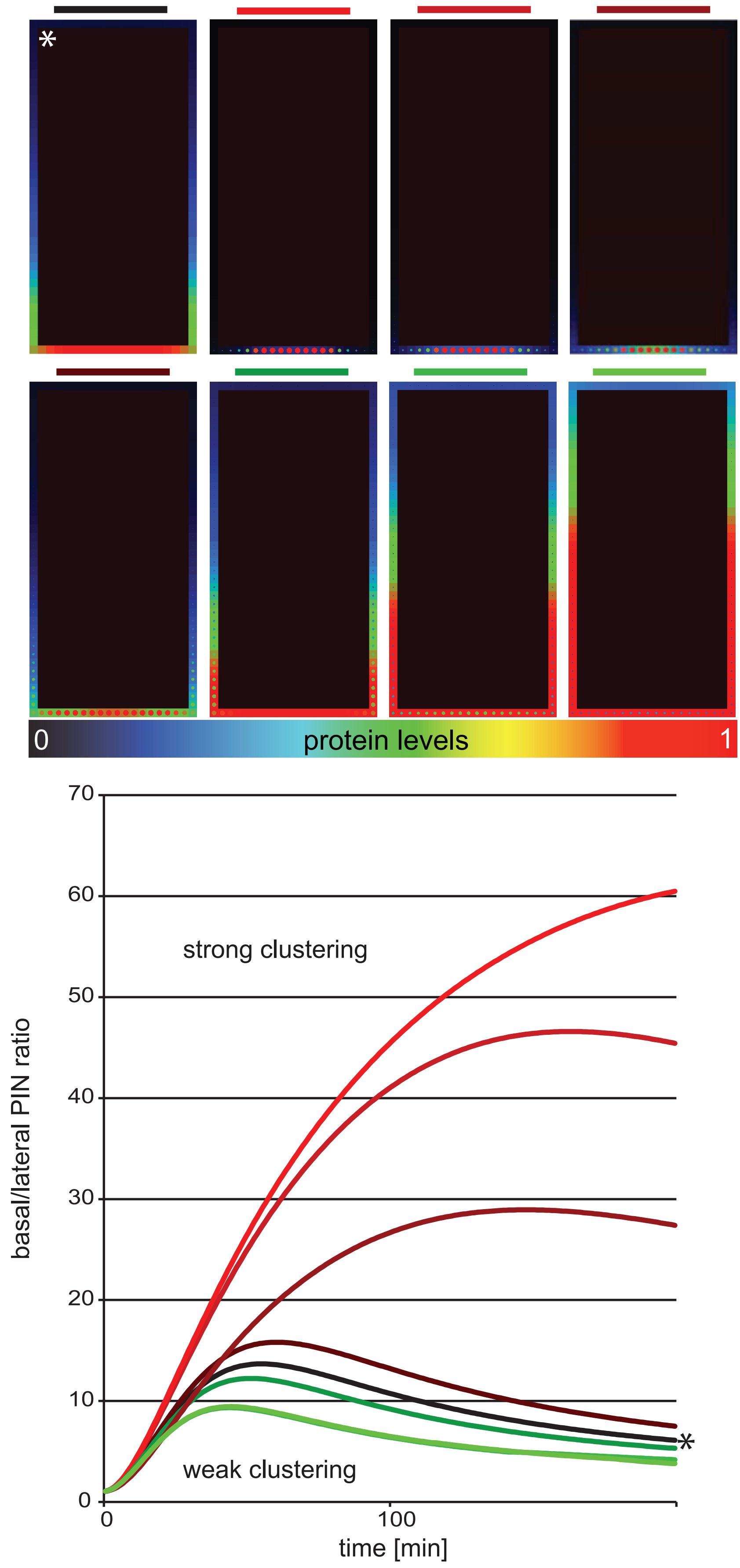

Supplementary Figure 15. In Silico Tests of Protein Clustering Impact on the Polarization Dynamics.
(A) Computational simulations representing predicted cell polarity that depends on two factors, lateral diffusion and polar recycling, in the non-polar secretion model (upper panel), and chart representing polarity index profiles (signal ratios between polar and non-polar domains) (lower panel). Since protein clustering may have an effect on lateral diffusion and total protein dynamics within the cells, we tested a broad range of parameters determining clustering degree (f1- non-mobile protein fraction). The black coloured line (asterisk) represents default clustering degree (f1 ~ 0.20) that was used in the polar secretion model. Color coding from red to light green represents clustering degree from high (f1 ~ 0.9) to low (f1 ~ 0.1), respectively (for the details see SupplementalSupplementary Methods). Protein levels are represented by color coding scheme, from low (0.0001) to high (1) (log scale).
